# Outer membrane vesicles as a platform for the discovery of antibodies to bacterial pathogens

**DOI:** 10.1007/s00253-024-13033-5

**Published:** 2024-02-24

**Authors:** Eric K. Lei, Aruba Azmat, Kevin A. Henry, Greg Hussack

**Affiliations:** 1 Human Health Therapeutics Research Centre, National Research Council Canada, Ottawa, ON Canada; 2 Department of Biochemistry, Microbiology and Immunology, University of Ottawa, Ottawa, ON Canada

**Keywords:** Antibody, Antimicrobial resistance, Immunization, Infectious disease, Integral membrane protein, Outer membrane vesicle

## Abstract

**Abstract:**

Bacterial outer membrane vesicles (OMVs) are nanosized spheroidal particles shed by gram-negative bacteria that contain biomolecules derived from the periplasmic space, the bacterial outer membrane, and possibly other compartments. OMVs can be purified from bacterial culture supernatants, and by genetically manipulating the bacterial cells that produce them, they can be engineered to harbor cargoes and/or display molecules of interest on their surfaces including antigens that are immunogenic in mammals. Since OMV bilayer-embedded components presumably maintain their native structures, OMVs may represent highly useful tools for generating antibodies to bacterial outer membrane targets. OMVs have historically been utilized as vaccines or vaccine constituents. Antibodies that target bacterial surfaces are increasingly being explored as antimicrobial agents either in unmodified form or as targeting moieties for bactericidal compounds. Here, we review the properties of OMVs, their use as immunogens, and their ability to elicit antibody responses against bacterial antigens. We highlight antigens from bacterial pathogens that have been successfully targeted using antibodies derived from OMV-based immunization and describe opportunities and limitations for OMVs as a platform for antimicrobial antibody development.

**Key points:**

• *Outer membrane vesicles (OMVs) of gram-negative bacteria bear cell-surface molecules*

• *OMV immunization allows rapid antibody (Ab) isolation to bacterial membrane targets*

• *Review and analysis of OMV-based immunogens for antimicrobial Ab development*

**Supplementary Information:**

The online version contains supplementary material available at 10.1007/s00253-024-13033-5.

## Introduction

The goal of this short review is to synthesize the results of studies that have used outer membrane vesicles (OMVs) from gram-negative bacteria as immunogens for the generation of antibodies (Abs) against bacterial cell-surface targets. We summarize the properties and natural functions of OMVs, common OMV sources, organisms and routes used for immunization, the types of anti-OMV Ab responses elicited (*e.g.*, polyclonal, monoclonal, isotypes), the properties of the target antigens, and the degree of characterization of the resulting Abs. Future perspectives regarding the opportunities and drawbacks of OMV-based Ab generation are presented. For comprehensive introductions to OMV biology, production, purification, characterization, and use in vaccine development, we direct readers to several other review articles (Balhuizen et al. 2021; Klimentova and Stulik 2015; Micoli and MacLennan 2020; Sartorio et al. 2021; Schwechheimer and Kuehn 2015).

## Properties of OMVs

**Fig. 1:**
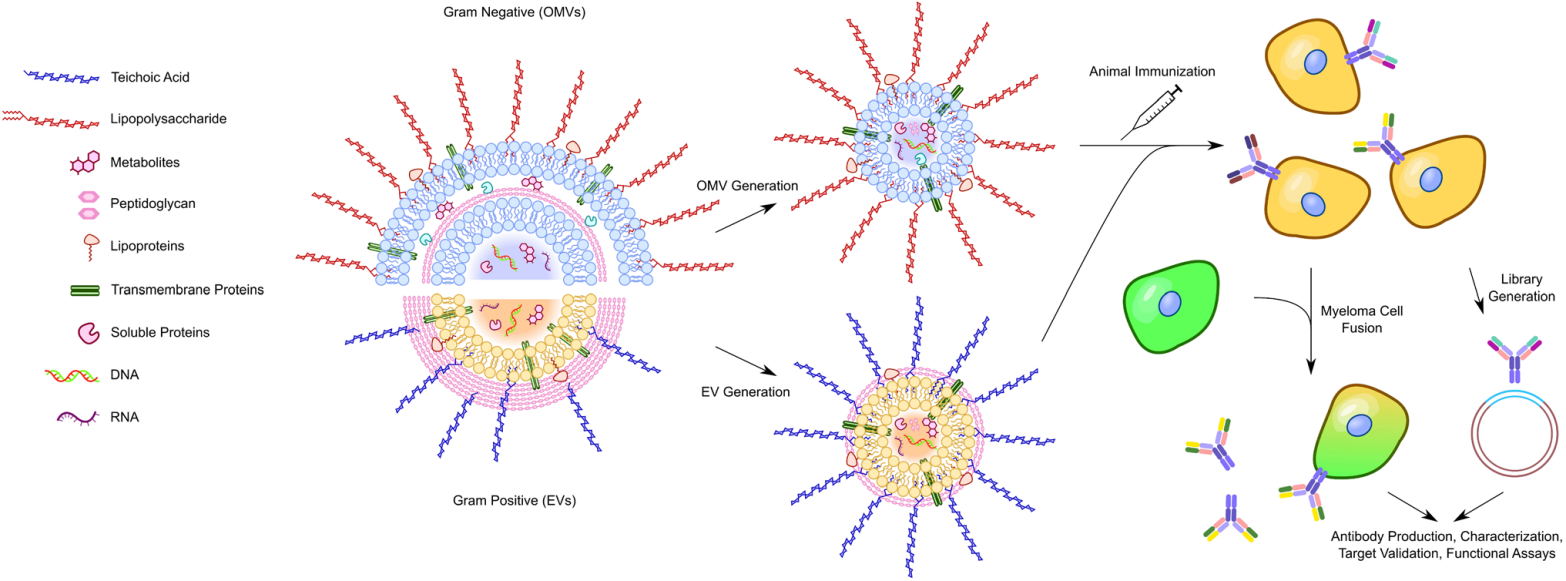
Schematic overview of antibody generation using outer membrane vesicles (OMVs) and extracellular vesicles (EVs). Purified OMVs from gram-negative bacteria or EVs from gram-positive bacteria are used as immunogens for animal immunizations (e.g., mouse, rat, chicken, rabbit, llama). Polyclonal antibodies against OMVs or bacterial antigens can be detected in the serum of responding animals. Hybridoma generation (myeloma cell fusion) or other selection approaches (e.g., phage display libraries) are used to isolate monoclonal antibodies (mAbs). The resulting mAbs are purified and characterized for affinity, specificity, and antimicrobial activity in functional assays. Target identification and validation, using techniques such as mass spectrometry and binding assays, reveal the antigen targeted by the mAb

OMVs are spheroidal particles 20 to 250 nm in diameter that are shed from the cell surfaces of nearly all gram-negative bacteria (Schwechheimer and Kuehn 2015). Similar particles shed by gram-positive bacteria and other microorganisms are generally referred to as membrane vesicles (MVs) or extracellular vesicles (EVs), hereafter termed EVs in this review (Fig. 1). OMVs are unilamellar vesicles bounded by lipids derived from the bacterial outer membrane (OM) and contain components derived from the periplasmic space as well as a subset of OM constituents, including lipoproteins, lipopolysaccharides (LPS), capsular polysaccharides, integral membrane proteins, and other OM-associated molecules (Lee et al. 2007; Murphy et al. 2014; Roier et al. 2015). The composition of the membrane-embedded and membrane-associated components of OMVs is closely related to that of the bacterial surface; however, the relative abundance of constituents can differ from the OM (Schwechheimer and Kuehn 2015) via mechanisms that are still unclear. Certain components can be enriched or depleted to varying degrees depending on environmental factors such as growth conditions, leading to heterogeneity among the OMVs shed from individual bacterial strains (Nagakubo et al. 2020). OMVs also carry a variety of cargoes in their luminal space including, with both periplasmic and cytoplasmic proteins, peptidoglycan, nucleic acids, and small molecule effectors involved in nutrient acquisition and signaling (Jan 2017). Like OM components, periplasmic components are enriched, depleted, or excluded in OMVs through poorly understood OMV biogenesis mechanisms (Bonnington and Kuehn 2014). The presence of cytoplasmic proteins in OMVs suggests the possibility of specific mechanisms of cellular transport and packaging, although none have yet been fully characterized. Note that evidence for the presence of cytoplasmic and inner membrane proteins in OMVs has been mostly based on proteomic analyses, some of which could not exclude the presence of lysed cells in the samples analyzed, and protein analyses have suggested that OMVs either lack or are highly depleted in these components depending on isolation or purification methodology (van de Waterbeemd et al. 2013). Similarly, although DNA has been detected in OMVs (Bitto et al. 2017), it has been suggested to be either or both surface-associated and luminally packaged, and in the former case, it remains unclear whether DNA originating from lysed cells may be present. The molecular mechanisms responsible for OMV shedding are only partially understood; however, the process of OMV formation is thought to begin with the detachment of peptidoglycan-associated OM proteins (Schwechheimer and Kuehn 2015). The inner leaflet of the OM contains anchored lipoproteins crosslinked to peptidoglycan; OMV formation is thought to occur when local crosslink levels decrease sufficiently (Schwechheimer et al. 2014). Additional factors such as the formation of lipid microdomains, accumulation of vesicle-facilitating molecules such as LPS subtypes, and the actions of quorum sensing molecules have also been suggested to contribute to the induction of membrane curvature, OMV formation, and budding (Schwechheimer and Kuehn 2015).

Gram-positive bacteria have also been shown to shed vesicles, which are referred to as EVs. The EVs of gram-positive bacteria are similar in size to OMVs but display cytoplasmic membrane components on their surfaces, have different phospholipid profiles, incorporate lipoteichoic acid and greater amounts of peptidoglycan, and harbor luminal components derived from the bacterial cytoplasm (Bose et al. 2020). Unlike the OMVs of gram-negative bacteria, gram-positive EVs are not anchored to an internal peptidoglycan layer but are instead anchored and physically blocked by the thick peptidoglycan-containing cell wall. Gram-positive bacterial cells induce localized disruption of the peptidoglycan layer, allowing for the passage of EVs through the resulting temporary channel (Bose et al. 2020). While less studied in comparison to gram-negative OMVs, gram-positive EVs are gaining interest due to their similar properties and applications as OMVs (Bose et al. 2020; Wang et al. 2018).

## Functions of OMVs

OMV release contributes to a wide array of biological functions in bacteria, with differences in OMV composition and function based on bacterial species (Jan 2017) and environmental conditions (Kulp and Kuehn 2010). OMVs directly contribute to bacterial survival and are generally used as long-distance effectors. OMV shedding allows for the removal of toxic or degraded cellular components, contributing to bacterial survival during cell stress (McBroom and Kuehn 2007). Bacteria can also use OMVs for nutrient acquisition from the environment, as they can contain hydrolytic enzymes, metal scavenging proteins, and lytic machinery (Schwechheimer and Kuehn 2015). OMVs released from bacterial pathogens can act directly as virulence factors by degrading host cellular and physical barriers (Stentz et al. 2018) and delivering toxins, virulence factors, or immune-modulating factors to host cells (Schwechheimer and Kuehn 2015). The physical presence of OMVs can also passively contribute to the degradation of antimicrobials via enzymatic activity (Ciofu et al. 2000) and/or adsorption of antimicrobials and bacteriophage that target OM components (Manning and Kuehn 2011). OMVs can also contain components that divert the complement system, contributing to the evasion of complement-mediated lysis (Dehinwal et al. 2021). They may also directly contribute to antimicrobial resistance development as vectors for horizontal gene transfer (Dell'Annunziata et al. 2021), although the mechanisms through which DNA is shuttled to OMVs remain unclear and the possibility of non-specific DNA association with OMV surfaces cannot be ruled out. OMVs also play a role in the development and restructuring of bacterial biofilms (Wang et al. 2015). As such, studies of OMVs may deepen our understanding of infections caused by gram-negative bacterial pathogens and offer new avenues for therapeutic targeting. Many of the functions of gram-negative OMVs are likely to extend to gram-positive EVs as well, although investigations of the functions of EVs are still in the early stages.

## Production and purification of OMVs

OMVs are an easy-to-produce source of non-replicative, presumably structurally preserved material derived from gram-negative bacterial cells and have been used extensively as antigens for immunization. Production of OMVs for research or therapy can be initiated using several different methodologies. OMVs produced by bacteria during growth without the addition of exogenous stimuli are referred to as spontaneous OMVs (sOMVs). They can be prepared by culturing bacteria and removing cells from the culture medium by centrifugation and filtration, followed by ultracentrifugation, ultrafiltration, or ammonium sulfate precipitation (Balhuizen et al. 2021; Wang et al. 2019a). These techniques can be expected to yield acellular material that contains OMVs as well as potentially other large extracellular materials such as flagella, fimbria, pili, and large protein complexes and/or aggregates. Higher OMV purity can be achieved by additional density gradient centrifugation and/or size exclusion chromatography steps; however, the presence of low levels of extracellular contaminants, some of which may be highly immunogenic, is generally difficult to rule out. Components derived from lysed cells may also contaminate OMV preparations. In some cases, the production of adequate amounts of OMVs may require enhancement of spontaneously occurring vesicle yields. OMV vesiculation can be promoted by the addition of mild non-ionic detergents to bacterial cultures, such as sodium deoxycholate or polyethylene glycol oleyl ether, which largely preserve vesicle integrity and protein folding (Gnopo et al. 2017; van der Pol et al. 2015). However, detergent-based OMVs (dOMVs) may have lower LPS content, less diverse phospholipid profiles, and altered proteomic profiles compared to sOMVs (van de Waterbeemd et al. 2010). Compared to sOMVs, dOMVs can also be less stable and more prone to aggregation (van de Waterbeemd et al. 2010). OMV vesiculation can also be induced through physical processes such as sonication, vortexing, pressure cell disruption, or by the addition of ethylenediaminetetraacetic acid (van der Pol et al. 2015). The resulting native OMVs (nOMVs) show more closely related proteomic profiles to sOMVs than dOMVs; however, differences in membrane protein composition between nOMVs and sOMVs are still observed (van de Waterbeemd et al. 2013; van de Waterbeemd et al. 2010). Interestingly, the composition of nOMVs is thought to more closely mirror that of the bacterial OM and periplasmic space compared with sOMVs (Gnopo et al. 2017; van der Pol et al. 2015). Both dOMVs and nOMVs contain higher levels of cytosolic proteins, including some that are not detected in sOMVs, possibly due to disruption of the inner membrane during mechanical or chemical induction of vesiculation (van de Waterbeemd et al. 2013). The attributes of OMVs produced using different strategies and their impacts on immunogenicity and antigen coverage may be desirable or detrimental depending on the application (van de Waterbeemd et al. 2013). Selection of a particular type of OMV depends on the recipient organism’s sensitivity to LPS, intended antigen coverage, required stability of the OMV formulation, and yield requirements.

## Genetic manipulation of OMVs

While the molecular mechanisms involved in OMV production are not fully understood, several pathways have been identified that allow for the modification of OMV vesiculation levels and composition. With respect to vesiculation levels, the deletion of OM proteins that associate with the peptidoglycan layer can result in disruption of peptidoglycan integrity, increased membrane stress, and enhanced vesiculation (Ojima et al. 2020). However, the impacts of mutations related to envelope stress and phospholipid accumulation on OMV composition are unknown. As the specific genes and proteins involved in OMV biogenesis differ between gram-negative bacteria, hypervesiculation mutation strategies are species specific. In *Escherichia coli*, mutation of genes encoding proteins that are involved in the linkages between the OM and inner membrane with peptidoglycan (*e.g.*, *tolA*, *tolB*, *ompC*, *ompF*, and *pal*) induces hypervesiculation (McBroom et al. 2006). Additionally, mutation of *nlpI*, a gene encoding a protein that restricts the activity of the peptidoglycan endopeptidase Spr, leads to reduced peptidoglycan crosslinking with the OM and hypervesiculation (Schwechheimer et al. 2015). Mutation of the *degP* gene encoding a periplasmic chaperone/protease increases vesiculation, as does mutation of the related stress signal transmission genes *degS* and *rseA*, presumably by increasing the accumulation of misfolded periplasmic proteins and, therefore, the need for export (McBroom et al. 2006). Studies of genetically induced hypervesiculation in various gram-negative pathogens are still in their infancy (Balhuizen et al. 2021). The available molecular toolbox for the control of OMV generation is likely to continue to expand as the molecular mechanisms of OMV biogenesis are further studied.

In addition to modulation of vesiculation levels, the composition of OMVs can be directly controlled through the manipulation of genes involved in OMV protein sorting. This was first shown in *E. coli* with the protein ClyA, which is concentrated into the membrane of OMVs (Wai et al. 2003) and can be used as a carrier for recombinantly fused antigens to the OMV surface (Kim et al. 2008). Subsequently, a number of other proteins (*e.g.*, *Neisseria meningitidis* fHbp (Salverda et al. 2016) and *E. coli* Hbp (Hays et al. 2018; Kuipers et al. 2015)) were shown to localize to OMVs and, via recombinant fusion, to present heterologous antigens on the vesicle surface in different gram-negative bacteria (Wang et al. 2019a). OMVs displaying heterologous fusions to OMV-associated membrane proteins on their surfaces have been found to elicit serum Ab responses in mice (Kuipers et al. 2015; Rappazzo et al. 2016; Salverda et al. 2016). Interestingly, luminal OMV cargo molecules can also elicit weaker Ab titers following immunization, a process that would be expected to require the release of OMV contents; however, few rigorous comparisons of the immunogenicity of surface-displayed *versus* luminal antigens have been conducted (Fantappie et al. 2014; Muralinath et al. 2011; Salverda et al. 2016). Alternatively, genes encoding proteins enriched in OMVs can be modified to include affinity tags such as SpyTag or avidin, and the presence of these handles on the OMV surface can be used to conjugate heterologous proteins and other molecules (Kim et al. 2008; Weyant et al. 2023). However, other factors such as heterologous protein complexity, fusion orientation, fusion protein expression levels, and potential impacts on bacterial growth and vesiculation rates must be considered in this process. It is expected that there would be size and complexity limitations on the types of heterologous proteins that can be successfully fused with OMV-resident proteins and presented on the OMV surface.

## Uses of OMVs as immunogens

OMVs have been developed as vaccines or vaccine components designed to protect against bacterial pathogens for several decades. The most well-studied bacterium in the context of OMV-based vaccines is *N. meningitidis* group B, with several products receiving regulatory approval in various jurisdictions (one, 4CMenB, by the FDA and EMA) since the 1980s. These include VA-MENGOC-BC^®^ (1989–present, Cuban National Immunization Program and elsewhere in Latin America and the Caribbean for epidemic control) and 4CMenB (Bexsero^®^; 2013–present, various jurisdictions including Europe and North America), a multicomponent vaccine containing three recombinant proteins produced in *E. coli* as well as the PorA-containing OMV preparation MeNZB™; the latter was used from 2004 to 2011 in New Zealand to control the spread of an epidemic strain (Micoli and MacLennan 2020). Bactericidal antibodies elicited by 4CMenB provide broad coverage (approximately 57–87%) against meningococcal serogroup B strains worldwide (Castilla et al. 2023). Another OMV-based vaccine developed in Norway in the 1980s, MenBVac^®^, formed the basis for the development of MeNZB™ and was used to control epidemic outbreaks in Norway and France. Both VA-MENGOC-BC^®^ and MeNZB™ are dOMV-based products extracted using deoxycholate. While these are the only examples of OMV vaccines that have received regulatory approval at the time of writing, several other OMV-based vaccines are under clinical development for pathogens including *Klebsiella pneumoniae, Salmonella* spp., *Shigella* spp., *Mycobacterium tuberculosis*, *Haemophilus* spp., and *Vibrio cholerae* (Micoli and MacLennan 2020). Preclinical investigations of OMV vaccines have included a wide range of targets derived from gram-negative and gram-positive bacteria as well as viruses (Lieberman 2022).

As bacterial resistance to the antibiotics currently used for human therapy continues to grow, the development of targeted Ab-based therapies may be necessary as an alternative. In this regard, OMV-based Ab development may provide a facile and rapid method for obtaining antibacterial Abs (Fig. 1). Given the success of OMV-based vaccines against *N. meningitidis*, researchers have analyzed the Ab responses elicited following OMV vaccination in a variety of animal models and in humans. It is assumed that the mechanisms underlying the generation of Ab responses to OMVs are similar to those for whole bacterial cells, involving uptake and processing by antigen-presenting cells followed by antigenic peptide presentation on MHC class II molecules to drive helper T cell responses, as well as surface capture of unprocessed opsonized OMV antigens for B-cell receptor engagement (Baker et al. 2021; Prior et al. 2021). Given that OMVs presumably present bacterial OM antigens in their native conformations, OMVs provide an attractive route for the development of Abs that recognize intact bacterial cells.

OMVs have been used extensively in generating polyclonal Abs (pAbs) against a variety of bacterial pathogens. Several studies have detected the presence of Abs to defined targets in the sera of immunized animals using enzyme-linked immunosorbent assay (ELISA), immunoprecipitation, western blotting, and mass spectrometry. The pAb responses observed in several of these studies suggest that monoclonal antibodies (mAbs) could potentially be developed against bacterial surface targets through immunization with OMVs. However, because pAb responses were detected in some cases to luminal or secreted proteins or to surface targets that are known to be shed, it remains unclear to what extent pAbs are generated against OMVs themselves (including integral OM proteins) *versus* copurified antigens and/or OMV degradation products. Note also that while some pAbs derived from OMV immunization have been assessed using in vitro functional assays (*e.g.*, bactericidal assays), it was not possible to ascribe functional activity to Abs directed against any specific antigen(s). A list of antigenic targets determined to be immunogenic following OMV or gram-positive EV-based immunizations is shown in Table 1 and Supplementary Table S1.

**Table 1:** Antigens eliciting a polyclonal antibody response following immunization with bacterial OMVs or EVs

Bacterium	Target class(es)	Antigen(s) recognized by pAbs^#^	Reference
*Acinetobacter baumannii*^a^	Integral OM protein, periplasmic protein, lipid-anchored IM protein	OmpP1/FadL/TodX family protein, OprB, OmpA, YbgF, PBP1b, CarO, putative exported protein, Omp25, Omp22, OmpW	Huang et al. (2016); Huang et al. (2019); McConnell et al. (2011)
*Bacillus anthracis*^b^	Secreted protein	PA^c^, LF^c^, EF^c^, ALO^c^	Rivera et al. (2010)
*Bordetella bronchiseptica*^*a*^, *Bordetella pertussis*^a^	OM glycolipid, surface-associated protein, cytoplasmic protein, fimbria, adhesin, secreted protein, integral OM protein	O-Ag^c^, lipid A-Kdo^c^, LPS^c^, BrkA, GroEL, Fim2^c^, Fim3^c^, FHA^c^, PRN^c^, PT^c^, BipA, Vag8	Bottero et al. (2018); Gasperini et al. (2018); Kanojia et al. (2018); Raeven et al. (2015); Raeven et al. (2016); Raeven et al. (2018); Raeven et al. (2020)
*Borrelia burgdorferi*^a^	Periplasmic and OM lipoprotein	OspA	Klouwens et al. (2021); Salverda et al. (2016)
*Burkholderia pseudomallei*^a^	OM glycolipid, surface-associated polysaccharide	LPS^c^, CPS^c^	Nieves et al. (2014); Petersen et al. (2014)
*Campylobacter jejuni*^*a*^	OM and periplasmic protein-associated polysaccharide	Heptapolysaccharide *N*-glycan	Price et al. (2016)
*Chlamydia muridarum*^a^	Integral OM protein, periplasmic protein	MOMP, HtrA	Bartolini et al. (2013); Weyant et al. (2023)
*Escherichia coli*^a^	OM glycolipid, surface-associated polysaccharide, integral OM protein, secreted protein, adhesin, flagellar component, pilin subunit, periplasmic protein	LPS^c^, PNAG^c^, LpxR, EtpA^c^, CexE^c^, LT^c^, FdeC, SslE^c^, FliC^c^, CfaB^c^, LPS^c^, PhoA	Hu et al. (2020); Leitner et al. (2015); Micoli et al. (2020); Noroozi et al. (2018); Rojas-Lopez et al. (2019); Roy et al. (2011); Schild et al. (2009); Stevenson et al. (2018); Wang et al. (2019b)
*Francisella tularensis*^*a*^	OM glycolipid	LPS^c^	Chen et al. (2016)
*Haemophilus influenzae*^a^	Surface-associated polysaccharide, integral OM protein, periplasmic protein	CPS^c^, Hup, BamA, HbpA, OmpP1, OmpP2, OmpP5, OmpP6	Micoli et al. (2020); Roier et al. (2012)
*Helicobacter pylori*^a^	OM lipoprotein	Lpp20^c^	Keenan et al. (2000)
*Mannheimia haemolytica*^*a*^	Secreted protein, integral OM protein	LKT^c^, SSA-1, TbpA, OmpD15, OmpP2, OmpA	Ayalew et al. (2013); Roier et al. (2013)
*Mycobacterium bovis*, *Mycobacterium tuberculosis*	Envelope glycolipid, surface-associated protein, secreted protein, periplasmic protein, cytoplasmic protein, outer leaflet lipoprotein, adhesin	LAM^c^, Acr^c^, Ag85B^c^, Mce1A, HBHA, RpIL (L7/L12), LpqH^c^, LppX^c^, VapC6, PstS1	Prados-Rosales et al. (2014); Reyes et al. (2013)
*Neisseria flavescens*^a^, *Neisseria lactamica*^a^, *Neisseria meningitidis*^a^, *Neisseria sicca*^a^	OM glycolipid, surface-associated polysaccharide, integral OM protein, periplasmic protein, adhesin, OM lipoprotein, cytoplasmic protein	LPS^c^, LOS^c^, CPS^c^, PorA, PorB, Opc, Rmp, RmpM, NspA, PilQ, OmpH, Omp85, MafA, OmpP1, LbpA, FrpB, NHBA^c^, fHbp^c^, NhhA, NadA, Opa, FetA, FbpA, BamA, MtrE, GroL, AceF, GuaB, NlpD	Alfini et al. (2022); Arigita et al. (2003); Bash et al. (2000); Beernink et al. (2012); Beernink et al. (2019a); Beernink et al. (2019b); Dalseg et al. (1999); Daniels-Treffandier et al. (2016); Devi et al. (1997); Fukasawa et al. (1999); Granoff et al. (2015); Haneberg et al. (1998); Hou et al. (2005); Keiser et al. (2011); Koeberling et al. (2007); Koeberling et al. (2008); Koeberling et al. (2009); Koeberling et al. (2011a); Koeberling et al. (2011b); Koeberling et al. (2014); Leduc et al. (2020); Marini et al. (2017); Martin et al. (2006); Matthias et al. (2020); Matthias et al. (2022); Micoli et al. (2020); Nagaputra et al. (2014); Necchi et al. (2021); Norheim et al. (2004); Norheim et al. (2005); Norheim et al. (2012); O’Dwyer et al. (2004); Pajon et al. (2013); Peak et al. (2013); Peeters et al. (1999); Perrett et al. (2015); Pettersson et al. (2006); Piccioli et al. (2023); Quakyi et al. (1999); Romeu et al. (2014); Sanders et al. (2015); Shoemaker et al. (2005); Troncoso et al. (2001); Trzewikoswki de Lima et al. (2020); Viviani et al. (2023); Wedege and Froholm (1986); Wedege et al. (2003); Wedege et al. (2007); Weynants et al. (2009); Williams et al. (2014); Zhang et al. (2016)
*Pasteurella multocida*^*a*^	Integral OM protein	OmpA, OmpH, OmpP6	Roier et al. (2013)
*Porphyromonas gingivalis*^*a*^	OM glycolipid	LPS^c^	Bai et al. (2015)
*Pseudomonas aeruginosa*^a^	Flagellin component, T3SS protein, secreted protein	FliC, PcrV^c^, HitA^c^	Li et al. (2021); Zhang et al. (2018)
*Salmonella enterica*^a^	OM glycolipid, surface-associated polysaccharide, integral OM protein, flagellar component, secreted protein	LPS^c^, CPS^c^, O-Ag^c^, Vi antigen^c^, OmpC, OmpF, OmpD, FliC^c^, SseB^c^, OmpA	Carvalho et al. (2019a); De Benedetto et al. (2017); Fiorino et al. (2021); Gasperini et al. (2021a); Gasperini et al. (2021b); Howlader et al. (2018); Liu et al. (2016); Liu et al. (2017); Micoli et al. (2018); Micoli et al. (2020); Muralinath et al. (2011); Necchi et al. (2021); Piccioli et al. (2022a); Schager et al. (2018); Sokaribo et al. (2021)
*Shigella boydii*^a^, *Shigella dysenteriae*^a^, *Shigella flexneri*^a^, *Shigella sonnei*^a^	OM glycolipid, integral OM and secreted protein, secreted protein, integral OM protein	LPS^c^, O-Ag^c^, VirG^c^, IpaB^c^, IpaC^c^, IpaD^c^, OmpA, OmpC, BamA, BamB, BamC, BamD	Arato et al. (2021); Frenck et al. (2021); Gasperini et al. (2021b); Gerke et al. (2015); Launay et al. (2017); Launay et al. (2019); Mancini et al. (2021); Mancini et al. (2023); Micoli et al. (2020); Micoli et al. (2021); Mitra et al. (2012); Mitra et al. (2013); Necchi et al. (2023); Obiero et al. (2017); Piccioli et al. (2022a); Raso et al. (2020); Richardson et al. (2021)
*Staphylococcus aureus*^b^	Secreted protein, IM lipoprotein, surface-associated and secreted protein	HLA^c^, LukE^c^, FhuD2, SpA^c^, EsxA^c^, Sbi^c^	Konig et al. (2021); Sun et al. (2023); Wang et al. (2018)
*Streptococcus mutans*^b^, *Streptococcus pneumoniae*^b^, *Streptococcus pyogenes*^b^, *Streptococcus suis*^b^	Surface-associated polysaccharide, cell wall-associated protein, cytoplasmic protein, cell wall-associated polysaccharide, secreted protein	CPS^c^, PspA, PLY^c^, GAC^c^, SaoA^c^, SAM_1372, SLO^c^, SpyCEP^c^, GftC^c^	Fantappie et al. (2014); Kuipers et al. (2015); Li et al. (2023); Muralinath et al. (2011); Nakamura et al. (2020); Nakao et al. (2022); Palmieri et al. (2022)
*Vibrio cholerae*^a^	OM glycolipid, secreted protein, pilus	LPS^c^, CTB^c^, TcpA	Adriani et al. (2018); Bishop et al. (2010); Leitner et al. (2013); Leitner et al. (2015); Perez et al. (2009)
*Yersinia pestis*^a^	T3SS protein, capsular protein	LcrV^c^, F1^c^	Carvalho et al. (2019b)

^#^See Supplementary Table S1 for immunogen and immunization method

^a^Gram-negative

^b^Gram-positive

^c^Secreted or shed molecule

*ALO*, anthrolysin O; *CPS*, capsular polysaccharide; *CTB*, cholera toxin B; *EF*, edema factor; *F1*, F1 capsular antigen; *FHA*, filamentous hemagglutinin; *fHbp*, factor H binding protein; *GAC*, group A carbohydrate; *HBHA*, heparin-binding hemagglutinin adhesin; *HLA*, alpha-hemolysin; *Hup*, haem-utilization protein; *IM*, inner membrane; *LAM*, lipoarabinomannan; *LF*, lethal factor; *LKT*, leukotoxin; *LOS*, lipooligosaccharide; *LPS*, lipopolysaccharide; *LT*, heat-labile toxin; *MOMP*, major outer membrane protein; *NHBA*, Neisserial heparin-binding antigen; *O-Ag*, O antigen; *OM*, outer membrane; *PA*, protective antigen; *PLY*, pneumolysin; *PNAG*, poly-N-acetyl-D-glucosamine; *PRN*, pertactin; *PT*, pertussis toxin; *Sbi*, *Staphylococcus aureus* binder of IgG; *SLO*, streptolysin O; *SpyCEP*, *Streptococcus* *pyogenes* cell-envelope proteinase; *SSA-1*, serotype 1-specific antigen; *T3SS*, type 3 secretion system

The use of OMVs derived from *Neisseria* species as immunogens has been studied extensively through efforts toward developing efficacious vaccines for meningitis. Ab responses following OMV immunization to the serotype-defining OM proteins (PorA, PorB, RmpM, Opa, and Opc), membrane polysaccharides, as well as many other surface-exposed virulence factors, have been detected and characterized (Awanye et al. 2019). While some serum pAb responses have been validated by ELISA using recombinant or purified antigens, many pAb responses have only been documented by western blot or immunoprecipitation; these techniques may be more susceptible to erroneous target attribution due to factors such as antigen abundance bias and low throughput, limiting the number of experimental controls that can be included. However, reproducible detection of the major OM protein classes, membrane-associated polysaccharides, and other associated antigens across independent experiments and in different organisms suggests that OMVs induce Ab responses to these antigens (Dalseg et al. 1999; Leduc et al. 2020; Viviani et al. 2023; Wedege et al. 2007). *Shigella* OMVs have also been well studied, and pAb responses to major OM antigens such as LPS, porins, and secretion system components have been observed (Mancini et al. 2021; Necchi et al. 2023). OMVs from several other gram-negative bacteria have been shown to induce pAb responses against membrane antigens (Table 1, Supplementary Table S1). Generation of pAbs to cell membrane targets of gram-positive pathogens such as *Bacillus anthracis*, *Staphylococcus aureus*, and *Streptococcus* species has also been demonstrated (Nakamura et al. 2020; Rivera et al. 2010; Wang et al. 2018). In some cases, pAbs to gram-positive antigens were obtained through direct immunization with EVs derived from the target bacteria; however, most pAbs were generated by heterologous expression of gram-positive bacterial antigens in OMVs derived from gram-negative bacteria, predominantly *E. coli* strains genetically modified for hypervesiculation.

## MAb generation using OMVs

OMV-based immunization has resulted in numerous target-validated mAbs primarily derived from mouse hybridomas (Table 2). Previous studies of *Neisseria* OMV-based vaccines have identified several OM proteins including PorB and RmpM as immunodominant antigens in mice and humans (Awanye et al. 2019). It is, therefore, unsurprising that OMV immunization enabled the isolation of mAbs directed to these two antigens. In two independent studies, mice were immunized with *N. meningitidis* strain 44/76 OMVs, and hybridoma-derived mAbs were analyzed by immunoblotting against OMVs as well as by epitope mapping using overlapping peptides from PorB (Delvig et al. 1995) and RmpM (Rosenqvist et al. 1999). This strategy allowed for the isolation of class 3 outer membrane protein (OMP; PorB) mAbs 188,C-1 (IgG3) and 152,D-8 (IgG1) (Delvig et al. 1995) as well as the class 4 OMP (Rmp) mAbs 155,B-4 (IgM), 173,G-1 (IgG1), and 185,H-8 (IgG2a) (Rosenqvist et al. 1999). Note that evidence for PorA binding by mAbs 188,C-1 and 152,D-8 was limited to equivocal ELISA binding to synthetic peptides, as well as potentially western blotting (although these results were not shown). Similarly, evidence for RmpM binding by mAbs 155,B-4, 173,G-1, and 185,H-8 was limited to ELISA binding to synthetic peptides whose locations in the protein’s three-dimensional structure were unclear, as well as potentially western blotting (although these results were not shown); none of the mAbs bound to live or killed bacterial cells by immunogold electron microscopy or flow cytometry. In addition to PorA and Rmp, mAbs to NspA were isolated using two separate methodologies. In one study, mice were sequentially immunized with OMVs derived from three strains of *N. meningitidis* with diverse serological classifications (M1090, BZ198, and Z1092) (Moe et al. 2002). Hybridoma supernatants were screened for bacterial cell binding and bactericidal activity, resulting in the discovery of mAb 14C7 (IgG3). In the same study, the mAb was validated in whole cell ELISA experiments using *E. coli* cells expressing *N. meningitidis* NspA. This effort built on earlier work from the same group in which mice were immunized with *E. coli* BL21(DE3) OMVs expressing *N. meningitidis* NspA, and the resulting mAbs (AL4, AL5, AL11, and AL1; all IgG2a) were validated by assessing ELISA binding to isogenic wild-type and NspA-knockout *Neisseria* cells (Moe et al. 2001). Two mAbs to *Neisseria* NadA (1079B6 and 4895F9, not isotyped) were also isolated by mouse OMV immunization, hybridoma generation, and immunoblotting. Subsequent antigen identification was conducted using mass spectrometry of tryptic digests (Fukasawa et al. 2003). In addition to protein targets, mAbs to *Neisseria* α2,8-linked polysialic acid have also been generated (Devi et al. 1996). To do so, OMVs from *N. meningitidis* M986-NCVl, a non-capsular mutant, were first purified and then chemically conjugated using adipic acid dihydrazide to *N*-deacetylated capsular polysaccharides (CPS) purified from *N. meningitidis* as well as *E. coli* K1 cells (Devi et al. 1996). Immunization and hybridoma generation yielded 11 mAbs of IgM and IgG isotypes, each of which bound to a subset of different *Neisseria* and *E. coli* CPS preparations. Interestingly, the binding of individual mAbs to polysialic acid preparations derived from the source bacterium by ELISA was affected by the immobilization method and/or protein conjugation status. Only three mAbs (A_2_, A_3_, and B_8_) showed significant bactericidal activity against *N. meningitidis.*

**Table 2:** MAbs obtained by immunization with bacterial OMVs

OMV source bacteria	Immunized organism	OMV type	Immunization schedule	Resulting mAb(s) and target	Validation technique(s)	Reference(s)
*Acinetobacter baumannii* ATCC 19606, ATCC 17978, ATCC 17961, and LAC-4	Llama	sOMV	s.c., 75 µg of each OMV with FCA on day 0, FIA on days 21, 28, and 35	VHH OMV81, binds CsuA/B	WB, ELISA, SPR, WC-ELISA, FL-MC	Lei et al. (2023)
*Escherichia coli* BL21(DE3) expressing recombinant *Neisseria meningitidis* NspA	CD-1 mouse	nOMV	s.c. first two injections then i.p. third injection, 10 ug OMV with CpG oligonucleotides on days 0, 21, and 42	IgG2a AL4, AL5, AL11, and AL12, bind NspA	ELISA, WC-ELISA, WC-FC, CDBA, passive transfer	Moe et al. (2001)
*Haemophilus influenzae* type b 26	BALB/c mouse	nOMV	i.p., 50 µg OMV with FCA on day 0, boosted with 30–40 µg at day 30, with a subset of animals boosted 30–40 µg at days 44–51 and 30–40 µg at days 58–65	mAb 6A2, binds LPS	IP, ELISA	Gulig and Hansen (1985); Robertson et al. (1982)
*Moraxella catarrhalis* 035E	BALB/c mouse	nOMV	i.p., 50 µg OMV with FCA at day 0, boost at day 28	IgG2a 10F3, binds CopB	WC-RIA, DB, WB, passive transfer	Helminen et al. (1993)
*Moraxella catarrhalis* O35E	BALB/c mouse	nOMV	i.p., 50 µg OMV with FCA at day 0, boost at day 28	IgG2a 17C7, binds UspA	WB, WC-RIA, passive transfer	Helminen et al. (1994)
*Neisseria meningitidis* M1090, BZ198, and Z1092	CD-1 mouse	nOMV	i.p., 5 ug OMV with alum on days 0, 21, and 42	IgG3 14C7, binds NspA	CDBA, passive transfer	Moe et al. (2002)
*Neisseria meningitidis* 44/76	BALB/c mouse	dOMV	s.c., 50 ug OMV in FCA on days 0 and 14	IgG3 188,C-1 and IgG1 152,D-8, bind class 3 OMP PorB	ELISA	Delvig et al. (1995)
*Neisseria meningitidis* 44/76-SL	BALB/c mouse	dOMV	s.c., 50 µg OMV with FCA on days 0 and 14	IgM 155,B-4, IgG1 173,G-1, and IgG2a 185,H-8, bind class 4 OMP RmpM	WB, WC-IG, WC-FC, CDBA, ELISA	Rosenqvist et al. (1999)
*Neisseria meningitidis* M986-NCVl with chemically conjugated *N. meningitidis* serogroup B and* Escherichia coli* K1 CPS	BALB/c mouse	sOMV	i.p., 5–10 µg of OMV-CPS conjugate with FCA on day 0, then FIA on day 28 for *E. coli* CPS conjugate, or with Ribi on days 0 and 21 for *N. meningitidis *CPS conjugate	Multiple mAbs binding subsets of *E. coli* K1 and K9 and *N. meningitidis* B CPS	ELISA, CDBA	Devi et al. (1996)
*Neisseria meningitidis* 3006 and M986	C3H/HePas mouse	sOMV	i.p., 17 µg OMV with alum on days 0, 14, and 28	mAbs 1079B6 and 4895F9, bind NadA	WB, MS, CDBA	Fukasawa et al. (2003)
*Porphyromonas gingivalis* TDC60	BALB/c mouse	sOMV	i.p.	mAb TDC4-33H, binds LPS	WB	Hijiya et al. (2010)
*Porphyromonas gingivalis* 381	BALB/c mouse	sOMV	i.p., 200 µg of OMV with FCA on days 0 and 14, i.v. with FCA on day 28	mAb Pg-Vc, binds hemagglutinin adhesin	WB, HAI	Shibata et al. (1998)
*Treponema pallidum*	BALB/c mouse	nOMV	s.c., 100 μL of OMV (5 × 10^10^ *T. pallidum* equivalents) with TiterMax at day 0, month 2, and month 4	mAb M131, binds phosphorylcholine	DB, WC-DB, WC-IG, FL-MC, CDBA	Blanco et al. (2005)

*CDBA*, complement-dependent bactericidal assay; *CPS*, capsular polysaccharide; *DB*, dot blot; *dOMV*, detergent-based outer membrane vesicle; *ELISA*, enzyme-linked immunosorbent assay; *FCA*, Freund’s complete adjuvant; *FIA*, Freund’s incomplete adjuvant; *FL-MC*, fluorescence microscopy; *HAI*, hemagglutinin inhibition assay;  *IP*, immunoprecipitation; *i.p.*, intraperitoneal; *i.v.*, intravenous; *MS*, mass spectrometry; *nOMV*, native outer membrane vesicle; *s.c.*, subcutaneous; *sOMV*, spontaneous outer membrane vesicle; *SPR*, surface plasmon resonance; *WB*, western blot; *WC-DB*, whole cell dot blot; *WC-ELISA*, whole cell enzyme-linked immunosorbent assay; *WC-FC*, whole cell flow cytometry; *WC-IG*, whole cell immunogold electron microscopy; *WC-RIA*, whole cell radioimmunoassay

In addition to *Neisseria*, OMVs have been effective antigenic sources in producing mAbs for several other bacterial pathogens. OMVs from *Haemophilus influenzae* type b were used to produce mAbs in mice via hybridoma generation (Robertson et al. 1982). The resulting mAbs were used to immunoprecipitate three OM protein antigens of different molecular weights. Only one mAb, 6A2 (not isotyped), was able to bind its cognate antigen on the surface of intact bacteria (Robertson et al. 1982). However, further experiments using denaturing western blots revealed that mAb 6A2 bound to *H. influenzae* LPS and that the erroneous immunoprecipitation result was due to the formation of a complex between LPS and an undefined OM protein, resulting in co-immunoprecipitation (Gulig and Hansen 1985). A similar strategy was used by the same group to identify several mAbs using *Moraxella catarrhalis* 035E OMVs, including mAb 10F3 (IgG2a) that was shown to bind CopB, an 80 kDa OM protein, by western blotting and colony blotting using whole *M. catarrhalis* cells and *E. coli* cells expressing *M. catarrhalis* CopB by western blotting (Helminen et al. 1993). The same approach was used to discover an anti-*M. catarrhalis* O35E mAb, 17C7 (IgG2a), that reacted with a single high molecular weight band in western blots and bound to whole bacterial cells. Antigen identification was conducted by plaque screening of a genomic *M. catarrhalis* O35E library constructed in a recombinant bacteriophage vector. Screening of bacteriophage plaques formed in *E. coli* cell lawns identified UspA as reactive with 17C7 by radiolabeling. MAb 17C7 was then found to bind UspA by western blot in both phage-infected *E. coli* cells expressing *M. catarrhalis* UspA and in *M. catarrhalis* O35E lysates (Helminen et al. 1994).

*Porphyromonas gingivalis* TDC60 OMVs purified via ammonium sulfate precipitation were used to immunize mice, and the resulting mAb TDC4-33H (not isotyped) potentially recognized *P. gingivalis* LPS based on ladder-like banding pattern in western blots and an ability to prevent IL-8 production by fibroblasts (Hijiya et al. 2010). *P. gingivalis* OMVs were also used to generate a mAb, Pg-Vc (not isotyped), against hemagglutinin (Shibata et al. 1998). The antigen was identified by western blotting against OMVs, followed by functional testing of inhibition of erythrocyte agglutination induced by OMVs. MAb Pg-Vc showed a similar banding pattern in western blots as an anti-hemagglutinating adhesion (HA-Ag2).

OMVs were prepared from *Treponema palladium* cells via disruption in a French pressure cell press, followed by purification through a continuous sugar gradient (Blanco et al. 2005). Mice were immunized with the OMV preparation, and sera were assessed for bactericidal activity. Hybridoma generation resulted in mAb M131 (IgM), which exhibited bactericidal activity through binding to a phosphorylcholine epitope specific to *T. pallidum*. Binding to whole cells was validated by dot-blot analysis and fluorescence microscopy.

Finally, *Acinetobacter baumannii* nOMVs were used as immunogens with the goal of generating OM protein-targeting single-domain Abs (VHHs) (Lei et al. 2023). Llama immunization with *A. baumannii* OMVs resulted in strong IgG1 and IgG2b pAb responses. Panning of a phage display library yielded a VHH, OMV81, whose target was identified as the OM pilin protein CsuA/B by western blotting and mass spectrometry. The VHH was demonstrated to bind intact *A. baumannii* cells by microscopy and whole cell ELISA.

In summary, OMV-based immunogens have been used to raise mAbs against targets that were subsequently identified through various techniques (Table 2). For some of these mAbs, robust binding data to native proteins and/or whole cells was not attempted or reported, and indirect validation techniques (*e.g.*, complement-dependent bactericidal assays, whole cell dot blot) were used to infer target antigen binding, while in other cases, the functional activity of the mAbs beyond antigen binding was not directly tested. In most of the cases highlighted here, passive immunization with mAbs was not attempted but is expected to be critical in demonstrating their utility as alternatives to conventional antimicrobials. Similar to pAbs (Table 1, Supplementary Table S1), several of the targets recognized by the mAbs could have been shed from degraded OMVs or copurified with OMVs, limiting confidence that the OMVs themselves acted as the primary immunogen. In addition, many of the mAb targets are amenable to recombinant overexpression or purification/extraction from bacteria and could thus likely have been generated using conventional immunization strategies.

## Perspectives on OMVs as immunogens

There are several opportunities and challenges in the use of gram-negative OMVs for the development of antimicrobial Abs. OMVs allow for the presentation of naturally OM-associated antigens to the immune system in a biologically relevant context without the need for complex purification and formulation processes. In this regard, they share similarities with live bacterial cells. OMVs are more convenient immunogens than bacterial cells due to their non-replicative nature, and because they lack the ability to cause infection in immunized animals, there are no concerns regarding gain of function gene transfer as in live cell immunizations. Interestingly, markers of immunogenicity such as dendritic cell activation and IgG titer have been found to be similar for OMVs and, in some cases, enhanced when compared to attenuated whole bacteria (Baker et al. 2021; Prior et al. 2021). For OMVs produced naturally by bacterial cells, factors such as endogenous protein folding, post-translational modification, oligomeric state, and binding interactions are expected to be largely preserved, whereas they may be compromised in a recombinant or purified antigen, an enriched OM protein preparation, or a killed bacterial cell preparation. It is less certain that chimeric exogenous antigens displayed on OMVs would maintain the same advantages as naturally OMV-associated antigens *versus* other types of immunogens. While using recombinant antigens for immunization and Ab screening makes target identification unnecessary, it introduces the potential for isolating Abs that fail to recognize native targets on live bacteria. Multiple-pass transmembrane proteins are important target antigens but are difficult to isolate and purify in their native conformations due to instability and aggregation propensity introduced by their transmembrane domains (Schlegel et al. 2014). Additionally, bacterial OMs are densely packed superstructures with a high degree of interaction between components. Isolation of antigens from the membrane architecture risks exposure of, and Ab generation to, inaccessible antigenic epitopes that would fail to be recognized by Abs on live whole cells. While the surfaces of OMVs may be imperfectly representative of the bacterial OM (van de Waterbeemd et al. 2013), they maintain some portion of the bacterial membrane architecture. This is especially important when considering antigens that have immunodominant epitopes that are sterically unavailable in a biological context, which would hinder the selection of Abs to subdominant but functional epitopes of non-native recombinant antigens. Additionally, OMV-based immunization may result in the discovery of unknown antigens or proteins which were not previously considered targets for Ab development. This ability may be important for rare and emerging bacterial pathogens for which extensive OM composition data are lacking.

In addition to reducing immune responses toward undesirable epitopes, OMV-based immunization can stimulate immune responses to weakly immunogenic epitopes through the presence of immunostimulatory molecules such as LPS (Piccioli et al. 2022b). As such, OMVs can be considered to naturally combine the functions of a lipid carrier and adjuvant. Studies comparing the immunogenicity of OMVs *versus* recombinant proteins combined with adjuvants have found similar or enhanced antibody titers using OMVs (Rosenthal et al. 2014; Rappazzo et al. 2016); however, additional studies are required to determine if the benefits of OMV immunizations are restricted to particular antigens and immunization strategies. For some applications (*e.g.*, human therapy), immunization with sOMVs may induce undesirably strong immune responses; however, immunogenicity can be decreased through formulation modifications or genetic manipulation of LPS content (Rossi et al. 2021). Immunization with OMVs through the intramuscular, intraperitoneal, and subcutaneous routes typically elicits high serum IgG, IgM, and IgA titers in a variety of animal models. Additionally, intranasal immunization may elicit stronger secretory IgA responses (*e.g.*, in saliva and the nasopharynx) with comparable serum titers (Gnopo et al. 2017).

While OMV-based immunization can be advantageous for rapid Ab discovery against OM targets, it also introduces challenges not present when using purified antigens. OMV membrane surfaces can retain soluble components originating from bacteria (*e.g.*, shed components such as flagella, fimbrae, and pilins, as well as potentially components derived from cell lysis) or other sources (Bitar et al. 2019), which could result in the generation of immune responses to components that are not naturally associated with OMVs or the OM (Klimentova and Stulik 2015). Because of this, additional purification steps after initial OMV isolation may be necessary, such as density gradient centrifugation and/or size exclusion chromatography. Even these steps may be insufficient to remove components that do not significantly differ in size or density from OMVs and/or strongly associate with OMVs in a non-specific manner. OMVs may also have stability considerations that complicate their use as immunogens compared to a purified antigen. Due to the presence of enzymatic components in OMVs, as well as the possibility of membrane fusion, aggregation, and/or precipitation, preparation of fresh OMVs may be necessary throughout the immunization and Ab generation process. For example, dOMVs are less stable and more prone to aggregation compared to other OMV classes (van de Waterbeemd et al. 2010). The generation of pAb responses following OMV immunization to luminal cargo proteins suggests that degradation and cargo release can occur; degradation may also be associated with the loss of native structure of OM proteins.

Another challenge for OMV-based immunization is that the immune sera may contain Abs to a variety of OM antigens, as well as potentially to membrane-associated and/or shed antigens, requiring mAbs to a particular antigen of interest to be isolated. Target validation using OMV immunization-derived mAbs is challenging but necessary. While it may be possible to select mAbs to a particular target without a purified antigen using genetic knockout or overexpressing cell lines, in many cases, it is more practical to generate a recombinant antigen for Ab screening. However, for difficult to purify antigens, Ab selection can be performed using the immunized OMV, with obtained mAb sequences analyzed for binding using western blot and mass spectrometry. Alternatively, a heterologous OMV or lipid carrier expressing the antigen of interest can be used to attempt to enrich for a defined antigen. While OMV immunization may be expected to result in the generation of Abs that bind OM targets on live cells, validation of cell binding using knockout and/or antigen-overexpressing strains, immunoprecipitation, or other complementary methods should be performed following antigen identification.

## Conclusions

OMVs and other types of bacterial EVs represent useful tools for the generation of Abs that targets the bacterial OM. In particular, they offer important theoretical advantages when attempting to generate Abs against challenging targets such as integral OM proteins; however, very few mAbs raised by OMV-based immunization to date have been conclusively shown to target these complex proteins in their native contexts. Challenges of OMV-based immunogens include potential copurification of immunogenic large extracellular material, non-specific association of soluble molecules with OMV surfaces, OMV degradation leading to loss of native OM architecture, and requirement for post-hoc antigen identification. The development of specific, high-affinity mAbs to bacterial membrane targets will be key to future efforts in antimicrobial drug development, diagnostic testing, and basic microbiology. As the development of antimicrobial resistance and epidemiological pressures increase the need for novel bacterial monitoring and treatment methods, Abs capable of targeting live bacteria are likely to be of increasing importance. OMV-based immunization, while in its relative infancy, fulfills a niche as it allows for rapid Ab generation against immunogenic OM targets, even for emerging or relatively unstudied pathogens.

## Supplementary information

Below is the link to the electronic supplementary material.

Supplementary file1 (PDF 373 KB)
