# Supplementary material for: Outer membrane vesicles as a platform for the discovery of antibodies to bacterial pathogens
Source: Appl Microbiol Biotechnol. 2024 Feb 24;108(1):232. doi: 10.1007/s00253-024-13033-5 (PMC10891261; doi:10.1007/s00253-024-13033-5)
Supplement: Supplementary file 1 — Supplementary file1 (PDF 373 KB) [file 253_2024_13033_MOESM1_ESM.pdf]

Applied Microbiology & Biotechnology

Supplemental Information

Outer membrane vesicles as a platform for the discovery of antibodies to bacterial pathogens

Eric K. Lei[1], Aruba Azmat[1], Kevin A. Henry[1,2], Greg Hussack[1,3]

[1]Human Health Therapeutics Research Centre, National Research Council Canada, Ottawa, Ontario, Canada

[2]Department of Biochemistry, Microbiology and Immunology, University of Ottawa, Ottawa, Ontario, Canada

[3]Correspondence: Greg.Hussack@nrc-cnrc.gc.ca

Supplemental Table S1 Polyclonal antibody responses generated by immunization with bacterial OMVs

**Bacteria and OMV source(s)**	**Organism(s) immunized**	**Immunization method(s)**	**pAb response(s) and target(s), if identified**	**Reference(s)**
Acinetobacter baumannii ATCC 19606	C57BL/6N mouse	s.c, 0.2 or 2 µg OMVs with 2 mg/mL alum adjuvant at 0, 2 and 4 weeks	Western blot evidence of IgG to OmpP1/FadL/ TodX family protein, OprB, OmpA, YbgF, PBP1b, CarO, putative exported protein, Omp25, Omp22 and OmpW	Huang et al. (2019)
Acinetobacter baumannii ATCC 19606	C57BL/6 mouse	i.m., 5 µg of OMVs with aluminum phosphate adjuvant on days 0 and 14	Measured IgG1 and IgG2 against *A. baumannii* OmpA, CarO, OmpW	McConnell et al. (2011)
Bacillus anthracis Sterne 34F2	BALB/c mouse	i.p., day 0 with FCA adjuvant and day 14 with FIA	Measured IgM against PA, LF, EF, ALO	Rivera et al. (2010)
Bacteroides thetaiotaomicron expressing chimeric *Yersinia pestis* LcrV or F1 fused to *B. thetaiotaomicron* OmpA leader	Rhesus macaque	i.n., with 12.5-50 µg of LcrV and F1 containing OMVs, or oral with 50 µg of OMVs on days 0 and 28	Detected IgG against F1, IgG and IgA against LcrV	Carvalho et al. (2019b)
Bacteroides thetaiotaomicron expressing chimeric *Salmonella enterica* ser. *Typhimurium* SL 1334 SseB or OmpA fused to *B. thetaiotaomicron* OmpA leader	C57BL/6 mouse	Oral or i.p. with 70 µg of OMV at months 0, 1 and 2, or i.n. with 70 µg of OMV at days 0, 14 and 21	Measured IgG and IgA titers against SseB and OmpA	Carvalho et al. (2019a)

Bordetella bronchiseptica 9.73	BALB/c mouse	s.c., on day 0 and 14	Detected IgG against *B. bronchiseptica* O-Ag and lipid A-Kdo	Bottero et al. (2018)
Bordetella pertussis B1917	BALB/c mouse	i.n. or s.c., 1-4 µg of OMV on days 0 and 28	Detected IgG, IgM and IgA against OMVs, *B. pertussis* B1917 cells, LPS, BrkA, GroEL, Fim2, Fim3, FHA, PRN, PT and Vag8 by western blot + mass spectrometry	Kanojia et al. (2018); Raeven et al. (2016); Raeven et al. (2018); Raeven et al. (2015); Raeven et al. (2020)
Bordetella pertussis W28 9K/129G	BALB/c mouse	i.p., 2.5 µg of OMV with aluminum hydroxide at weeks 0, 4 and 8	Detected IgG binding to recombinant BrkA, Vag8 and BipA expressed on *Escherichia coli* cells	Gasperini et al. (2018)
Burkholderia pseudomallei 1026b	BALB/c mouse	s.c., 5 µg of OMV at days 0, 21 and 42	Detected IgM, IgG1, IgG2a and IgG3 against OMV, LPS and CPS	Nieves et al. (2014)
Burkholderia pseudomallei 1026b	Rhesus macaque	s.c., 25 µg, 50 µg, and 100 µg of OMV with 400 µg of CpG adjuvant on days 0, 28 and 56, respectively	Measured IgG against LPS and CPS	Petersen et al. (2014)
Enterotoxigenic *Escherichia coli*	CD1 mouse	i.n., 20 µg OMV on days 0, 14 and 28	Detected IgG against recombinant EtpA, CexE and LT on western blot	Roy et al. (2011)
Enterotoxigenic *Escherichia coli,* chitosan encapsulated	BALB/c mouse	s.c., 10 µg OMV or oral, 50 µg	Measured IgG against LT subunit B	Noroozi et al. (2018)
Escherichia coli O1, O2 and O78	Arbor Acres broiler chicken	i.m., 10, 50 and 100 µg of OMVs on days 0, 7 and 14	Detected IgG against OMVs and LPS	Hu et al. (2020)

Escherichia coli O78	Lohmann chicken	i.m., 75 µg OMVs at days 0 and 14	Detected IgG against LPS	Wang et al. (2019)
Escherichia coli BL21(DE3) expressing chimeric *Chlamydia muridarum* HtrA fused to *E. coli* OmpA leader	BALB/c mouse	i.m., 50 µg OMV with aluminum hydroxide at weeks 0, 2 and 4	Detected IgG against HtrA-OMV and recombinant HtrA, confirmed IgG binding to *C. muridarum* elementary bodies	Bartolini et al. (2013)
Escherichia coli BL21(DE3) expressing chimeric *Streptococcus pyogenes* SLO, Bla, SpyCEP, Spy0269 and SAM_1372 fused to *E. coli* OmpA leader	CD1 mouse	i.p., 25 µg of OMV on days 0, 21 and 35	Measured IgG1 and IgG2 against *S. pyogenes* SLO, SpyCEP and SAM_1372	Fantappie et al. (2014)
Escherichia coli BL21(DE3)Δ60 expressing chimeric *Staphylococcus aureus* FhuD2- HLA_{H35L}, $ClfA_{Y338A}$- LukE and SpA_{KKAA}-HLA_{H35L} fused to an *E. coli* Lpp leader	CD1 mouse	i.p. and i.m., 20 µg of OMV with alum on days 0, 14 and 28	Detected IgG against *S. aureus* FhuD2, LukE, SpA and HLA	Konig et al. (2021)
Escherichia coli CLM37 displaying *Streptococcus pneumoniae* CPS serotype 14 or *Campylobacter jejuni* heptapolysaccharide *N*-glycan	BALB/c mouse for OMVs displaying *S. pneumoniae* CPS<br><br>Leghorn chicken for OMVs displaying *C. jejuni* heptapolysaccharide *N*-glycan	Mice: i.p., 2 µg of OMV at days 0, 14 and 21<br><br>Chickens: oral, 0.5 µg of OMV days 0 and 14	Detected mouse IgG against whole cell *S. pneumoniae* serotype 14 and chicken serum IgY against BSA conjugated to *C. jejuni* heptapolysaccharide *N*-glycan	Price et al. (2016)

Escherichia coli DH5α expressing chimeric *Acinetobacter baumannii* ATCC 17978 Omp22 fused to *E*. *coli* W-15 ClyA	ICR mouse	s.c., 5-50 µg of Omp22-ClyA containing OMV or wtOMV on days 0 and 14	Measured IgG against OMVs and Omp22	Huang et al. (2016)
Escherichia coli JC8031 displaying *Francisella tularensis* subsp. *holarctica* O-Ag	BALB/c mouse	i.p., 10 µg OMV on days 0, 14 and 28	Detected IgG against *F. tularensis* LPS	Chen et al. (2016)
Escherichia coli JC8031 displaying *E. coli* PNAG, deacetylated PNAG	BALB/c mouse	s.c., 10 µg OMV on weeks 0, 3 and 6	Detected IgG and IgM against PNAG	Stevenson et al. (2018)
Escherichia coli K12 expressing *E. coli* MC001 LpxR	BALB/c mouse	i.p., 10 µg OMV with alum on day 0, 5 µg OMV on days 20 and 34	Detected IgG against *E. coli* MC001 LpxR	Rojas-Lopez et al. (2019)
Escherichia coli KPM404 expressing chimeric *E. coli* Lpp-OmpA-enhanced monoavidin; OMVs conjugated to GFP or *Chlamydia muridarum* MOMP	BALB/c mouse	s.c., 20 µg of OMV on days 0, 21 and 42	Detected IgG against GFP and MOMP	Weyant et al. (2023)
Escherichia coli MG1655 expressing chimeric *E. coli* $OmpA_{1\text{-}155}$-SpyCatcher; OMVs conjugated to chimeric *Staphylococcus aureus* SpyTag-EsxA, SpyTag-Sbi or SpyTag-SpA	BALB/c mouse	s.c., 50 µg of OMV conjugate on days 0, 14 and 28	Detected IgG against *S. aureus* EsxA, Sbi and SpA	Sun et al. (2023)
Escherichia coli Nissle 1917 displaying serotype	BALB/c mouse	s.c., 1 µg OMV on days 0 and 21	Detected IgG and IgM against serotype 14 CPS	Nakao et al. (2022)

14 *Streptococcus pneumoniae* CPS				
Haemophilus influenzae 019-R	BALB/c mouse	i.n. or i.p., 2-25 µg OMV on days 0, 14 and 28	Detected IgG against Hup, BamA, HbpA, OmpP1, OmpP2, OmpP5 and OmpP6	Roier et al. (2012)
Helicobacter pylori 60190	BALB/c mouse	Oral, 50 µg OMV with CTB adjuvant on days 0, 7, 14 and 21	Detected IgG against Lpp20	Keenan et al. (2000)
Mannheimia haemolytica 89010807N	BALB/c mouse<br><br>Holstein calf	s.c., 10-50 µg OMV with or without FIA on days 0 and 14 for mice, 150 µg OMV with FIA for calves	Detected IgG against LKT in both animals	Ayalew et al. (2013)
Mycobacterium bovis BCG	BALB/c mouse	i.p., 50 mg proteoliposome with FIA at days 0 and 21	Detected IgG against BCG whole cells, *M. bovis* cell wall fractions, soluble cell wall proteins, LAM, Acr, Ag85B, Mce1A, HBHA and RplL (L7/L12)	Reyes et al. (2013)
Mycobacterium bovis BCG<br><br>*Mycobacterium tuberculosis* H37Rv	C57BL/6 mouse	s.c., 2.5 µg of either OMV on days 0 and 21	Detected IgG and IgM to *M. tuberculosis* H37Rv protein extracts, identified LpqH, LppX, VapC6 and PstS1	Prados-Rosales et al. (2014)
Neisseria flavescens 2830 expressing *Neisseria meningitidis* NspA	NIH mouse	s.c., 10 µg of OMV on days 0, 20 and 27	Detected IgG against NspA	O'Dwyer et al. (2004)
Neisseria lactamica NIP3 and NIP2<br><br>*Neisseria sicca* NsP1 and NsP3	BALB/c mouse	i.p., 20 µg OMV with FCA on day 0, FIA on day 14 and no adjuvant on day 28	Detected IgG against RmpM	Troncoso et al. (2001)

Neisseria meningitidis NZ98/254<br><br>*Escherichia coli* BL21(DE3) expressing chimeric *N. meningitidis* PorA, Opc, NspA, PilQ, OmpH, Omp85, MafA, OmpP1, LbpA, or FrpB fused to *E. coli* OmpA leader	CD1 mouse	i.p., 8 µg of OMV with aluminum hydroxide adjuvant on days 0, 21 and 35	Detected IgG against PorA, Opc, NspA, PilQ, OmpH, Omp85, MafA, OmpP1, LbpA and FrpB	Viviani et al. (2023)
Neisseria meningitidis NZ98/254	Human	1 dose of 4CMenB on days 0 and 28	Detected IgG against NHBA	Perrett et al. (2015)
Neisseria meningitidis (multiple strains)	CD1 mouse	i.p., 1.25-5 µg OMV with alum adjuvant on days 0, 21 and 42	Detected IgG and IgM against GNA1870 fHbp	Hou et al. (2005); Koeberling et al. (2007)
Neisseria meningitidis (multiple strains)	OF-1 mouse	i.m., 10 µg of OMV with 100 µg of aluminum hydroxide on days 0, 21 and 28	Detected IgG against NhhA derived from strain H44/76	Peak et al. (2013)
Neisseria meningitidis (multiple strains)	BALB/c and CD-1 mice<br><br>Rhesus macaque	i.p., 2.5 µg of OMV with alum adjuvant on days 0 and 21 for BALB/c mice<br><br>i.p., 2-5 µg of OMV with alum adjuvant on days 0, 21 and 42 for CD-1 mice<br><br>i.m., 25 µg of OMV with alum adjuvant on days 0 and 42 for macaques	Detected IgG in all immunized animals against NadA, fHbp v1 and v2, and LOS	Beernink et al. (2019a); Beernink et al. (2012); Beernink et al. (2019b); Granoff et al. (2015); Koeberling et al. (2011a); Koeberling et

				al. (2009); Koeberling et al. (2008); Koeberling et al. (2011b); Pajon et al. (2013)
Neisseria meningitidis 44/76	OF1 mouse	i.m., 5 µg OMV on days 0, 21 and 28	Detected IgG against LOS, specifically the LNnT epitope	Weynants et al. (2009)
Neisseria meningitidis 44/76	NIH/RIVM mouse	s.c., 0.33-10 µg of OMV on days 0 and 28	Detected IgG against PorA, Rmp, Opa/Opc and a 20 kDa OMP	Peeters et al. (1999)
Neisseria meningitidis 44/76	BALB/c mouse	i.n. or rectal, 25-250 µg OMV with CTB or FIA adjuvant on days 0, 7, 14 and 21	Detected IgG and IgA against PorA, PorB, RmpM and Opc	Dalseg et al. (1999)
Neisseria meningitidis 44/76	NIH/OlaHSD mouse	s.c., 2.5 µg of OMV on days 0 and 28	Detected IgG against PorA	Nagaputra et al. (2014)
Neisseria meningitidis 44/76	Human	i.m., 25 µg of OMV with alum on months 0, 2 and 4	Detected IgG against PorA, PorB, Opc and Opa	Wedege et al. (2003)
Neisseria meningitidis 44/76	Human	i.n., 250 µg OMV on days 0, 7, 14 and 21	Detected IgG and IgA against LPS, PorA, PorB, RmpM and Opc	Haneberg et al. (1998)
Neisseria meningitidis 44/76, 8570 and B16B6	CD1 mouse<br><br>Rhesus macaque	i.m., 1-3 µg OMV on days 0 and 14 for mice, 12-37.5 µg OMV on days 0, 56 and 112 for macaques	Detected IgG against fHbp v1, fHbp v2 and PorA in mice and rhesus macaques	Zhang et al. (2016)
Neisseria meningitidis 44/76-SL or NZ98/254	Human	i.m., 25-50 µg of OMV from NZ98/254 or 25 µg	Detected IgG against Omp85, FetA, PorA, PorB, FbpA, RmpM, Opc, NspA and LPS	Wedege et al. (2007)

		of OMV from 44/76-SL on days 0, 42 and 84		
Neisseria meningitidis 8750	Human	i.m., 25-75 µg of OMV at days 0, 42 and 84	Detected IgG against OMV, Opc, fHbp and LPS	Keiser et al. (2011)
Neisseria meningitidis 9162	New Zealand White rabbit	i.m. or oral, 300 µg of OMV on day 0	Detected IgG against LOS	Shoemaker et al. (2005)
Neisseria meningitidis 99M, M986 OMVs conjugated to CPS of group B *N. meningitidis*	Rhesus macaque	i.m., 5 µg of OMV conjugate in MPL+TDM on days 0, 42 and 98	Detected IgG and IgM against CPS of group B *N. meningitidis*	Devi et al. (1997)
Neisseria meningitidis B:4:P1.9, B:8:P1.6	BALB/c, A/Sn mouse	s.c., 2 µg of OMV on days 0, 20 and 35	Detected IgG against PorA and Opa	Trzewikoswki de Lima et al. (2020)
Neisseria meningitidis CH501, Cu385 or H355	New Zealand White rabbit	i.m., 25 µg OMV on months 0, 1 and either 2 or 6	Detected IgGs against PorB by western blot	Bash et al. (2000)
Neisseria meningitidis F91	BALB/c mouse	s.c., 10 µg of OMVs measured on days 0, 14 and 28	Detected IgG against PorA	Arigita et al. (2003)
Neisseria meningitidis H44/76 SMenPF1.2	NIH/OlaHsd mouse<br><br>New Zealand White rabbit	For mice, s.c., 1 to 10 µg of OMVs with alum adjuvant on days 0, 21, 42 and 63<br><br>For rabbits, s.c., 25 µg of OMVs with alum adjuvant on days 0 and 21	Detected mouse and rabbit IgG against OMVs, PorA and FetA	Sanders et al. (2015)
Neisseria meningitidis H44/76 ST32 complex	Human	i.m., 25 to 50 µg of OMV on day 0, 21 and 42	Detected IgG against several *N. meningitidis* proteins *via* 2D western blot + mass spectrometry	Williams et al. (2014)

Neisseria meningitidis H44/76Lpxl1	C57BL/6 mouse	i.m., 2.5 or 5 µg of OMVs with alum adjuvant on days 0 and 28	Detected mouse IgG against OMVs, *N. meningitidis* cells, fHbp and PorA	Daniels-Treffandier et al. (2016)
Neisseria meningitidis HB-1 expressing chimeric *Borrelia burgdorferi 297* OspA fused to truncated forms of *N. meningitidis* fHbp	BALB/cOlaHsd mouse	s.c., 1 or 4 µg OMVs at days 0 and 28	Detected IgG against *B. burgdorferi* OspA	Salverda et al. (2016)
Neisseria meningitidis M986	C57BL/6 mouse	i.p., 75 µg of LOS in OMVs on days 0, 1 and 2	Detected IgG against LOS	Quakyi et al. (1999)
Neisseria meningitidis M986 NCV-1 serotype 2a	Human	50 ug of OMV on days 0 and 28	Detected IgG, IgA and IgM against PorA and several unidentified proteins by western blot	Wedege and Froholm (1986)
Neisseria meningitidis MC58	BALB/c mouse	i.p. or s.c. on days 0, 21 and 42	Detected cross reactive IgG against *Neisseria gonorrhoeae* PilQ, BamA, MtrE, NHBA, PorB and Opa by western blot + mass spectrometry	Leduc et al. (2020)
Neisseria meningitidis MC58	BALB/cAnNCr mouse	i.p., 12.5 µg OMV with alum on days 0, 14 and 28	Detected IgG against PorA, PorB, RmpM and LOS	Matthias et al. (2020)
Neisseria meningitidis MC58	BALB/cAnNCr mouse	i.p., 12.5 µg OMV with alum on days 0, 14 and 28	Detected IgG against PorB, Rmp, PilQ, MtrE, GroL, AceF, GuaB and NlpD by co-immunoprecipitation and mass spectrometry	Matthias et al. (2022)
Neisseria meningitidis Mk 83/94, F8238, Mk 686/02, Mk 760/02, Mk196/02 and Mk222/02	BomTac:NMRI mouse	s.c., 1-10 µg of OMV on days 0 and 21	Detected IgG against *N. meningitidis* F8238 OMVs, Mk 686/02 live cells, NspA by ELISA, PorA, PorB, NadA, RmpM, Opc, Opa and LOS	Norheim et al. (2005); Norheim et al. (2004);

				Norheim et al. (2012)
Neisseria meningitidis Mk499/03 and Mk222/02	BALB/c mouse	s.c., 2.5 µg of each strain of OMV with 5 µg PSA, adsorbed to alum adjuvant on day 0 and 21	Detected IgG against *N. meningitidis* CPS	Romeu et al. (2014)
Neisseria meningitidis serogroup B conjugated to fHbp variant 3	C57BL/6 and CD1 mice	i.m. or i.p., 0.625-100 µg OMV with or without alum adjuvant on days 0, 21 and 35	Detected IgG1, IgG2a, IgG2b and IgG3 against fHbp	Alfini et al. (2022); Piccioli et al. (2023)
Neisseria meningitidis serogroup B expressing chimeric *Borrelia burgdorferi* B31 OspA fused to truncated *N. meningitidis* fHbp	C3H/HeN mouse	40 µg of OMVs at days 0, 14 and 28	Measured IgG1 and IgG2a against *B. burgdorferi* OspA	Klouwens et al. (2021)
Neisseria meningitidis serogroup B NZ98/254	Human	i.m., 25-50 µg of OMV at weeks 0, 6 and 12	Detected IgG against OMV antigens by western blot, validated binding to PorA by mutational analysis	Martin et al. (2006)
Neisseria meningitidis serogroup W	CD-1 mouse	i.p., 0.2 to 5 µg of OMV with alum adjuvant on days 0, 14 and 28 or days 0 and 28	Detected IgG against fHbp	Koeberling et al. (2014); Marini et al. (2017)
Neisseria meningitidis H44/76 CE2001	OF-1 mouse	i.m., 5 µg OMV with alum adjuvant on days 0, 21 and 35	Detected IgG against LbpA	Pettersson et al. (2006)
Neisseria meningitidis N44/89 conjugated to *N. meningitidis* serogroup C CPS	C3H/HePas mouse	i.p., 2.5 µg of CPS on OMVs with alum adjuvant on days 0, 14 and 28	Detected IgG against serogroup C CPS and PorA by western blot	Fukasawa et al. (1999)

Pasteurella multocida P4881<br><br>*Mannheimia haemolytica* SH789	BALB/c mouse	i.n., 25 µg OMV on days 0, 14 and 28	Detected IgA and IgG against OMVs from *P. multocida* and *M. haemolytica*. Immunoprecipitation using pooled sera from *P. multocida* OMVs identified outer membrane proteins OmpA, OmpH and P6. Pooled sera from *M. haemolytica* OMVs identified SSA-1, TbpA, OmpD15, OmpP2 and OmpA	Roier et al. (2013)
Porphyromonas gingivalis ATCC 33277	BALB/c mouse	i.n.,1 µg of OMVs and 10 µg of poly(I:C) on weeks 0 and 3	Detected IgG against *P. gingivalis* LPS and whole cells	Bai et al. (2015)
Pseudomonas aeruginosa PAO1	BALB/c mouse	i.m., 30 µg of OMV and 30 µg aluminum phosphate adjuvant on days 0, 14 and 21	Detected IgG1 and IgG2a against *P. aeruginosa* PAO1 OMVs, cell lysates and FliC	Zhang et al. (2018)
Salmonella enterica ser. Paratyphi A NVGH308 displaying *Salmonella Typhi* Vi antigen	C57BL/6 mouse	s.c., OMV containing ~5 µg of O-Ag and ~5 µg of Vi antigen at days 0 and 28	Detected IgG against Vi antigen as well as *S. enterica* Paratyphi A O:2 and O9 O-Ags	Gasperini et al. (2021a)
Salmonella enterica ser. Typhimurium 1418 *ΔtolR* conjugated to *Neisseria meningitidis* MenB fHbp, MenC or MenA oligosaccharides<br><br>*N. meningitidis* Men B ΔsynX, Δctra, Δgna33, ΔlpxL1 conjugated to MenC or MenA oligosaccharides, *Haemophilus influenzae* type b oligosaccharides	CD1 mouse	s.c. or i.m., OMV conjugates with aluminum hydroxide on days 0 and 28<br><br>*S. enterica* OMVs: 0.75 µg fHbp, 1 µg MenC and MenA oligosaccharides<br><br>*N. meningitidis* OMVs: 1 µg MenC, MenB, or *H. influenzae* type b oligosaccharides	Detected IgG against *Salmonella* O-Ag, *N. meningitidis* fHbp, MenA, MenC, MenC polysaccharide, *H. influenzae* polysaccharide, *E. coli* FdeC and SslE, and *S. sonnei* LPS	Micoli et al. (2020)

Shigella sonnei ΔtolR ΔvirG ΔhtrB 53G conjugated to *Escherichia coli* SslE and/or FdeC		*S. sonnei* OMVs: 5 µg FdeC, 5 µg SslE, or 5 µg FdeC + SslE		
Salmonella enterica ser. Typhimurium SGSC1418, *S. enterica* ser. Enteritidis SA618, genetically modified for hypervesiculation (ΔtolR) and to reduce LPS reactogenicity (ΔmsbB, ΔhtrB and ΔpagP)	CD1 mouse	s.c., OMV containing 1 µg of O-Ag with Alhydrogel adjuvant on days 0 and 28	Measured IgG against O-Ag	De Benedetto et al. (2017)
Salmonella enterica ser. Typhimurium SL3261 ΔtolRA expressing chimeric truncated *Escherichia coli* HbpD fused to truncated *Streptococcus pneumoniae* PspA or PLY	BALB/c mouse	i.n., 4 µg of OMV in 1-3 doses at days 0, 14 and 28	Detected IgG and IgA against *S. pneumoniae* PspA and PLY	Kuipers et al. (2015)
Salmonella enterica ser. Typhimurium χ9281 expressing chimeric *Streptococcus pneumoniae* PsP fused to a beta-lactamase signal sequence	BALB/c mouse	i.n., 50 µg of OMV at weeks 0, 1, 2 and 3	Detected IgG against OMVs, *Salmonella* LPS, *Salmonella* outer membrane protein extracts and *S. pneumoniae* PspA	Muralinath et al. (2011)
Salmonella enterica Enteritidis χ3744	BALB/c mouse	i.n. with 20 µg OMV or i.p. with 5 µg on days 0 and 30	Measured IgG and IgA against OMVs and LPS	Liu et al. (2017)

Salmonella enterica var. Typhimurium SL1418 ΔtolR	C57BL/6 mouse	i.p., 1 µg of OMV	Detected IgG and IgM against OMVs, LPS, OmpC, OmpF and OmpD	Schager et al. (2018)
Salmonella enterica Typhi C-6953<br><br>*S. enterica* Paratyphi A C-6915	BALB/c mouse	Oral, 25 µg of OMVs on days 0, 14 and 28	Detected IgG, IgA and IgM against OMVs, measured IgG against LPS	Howlader et al. (2018)
Salmonella enterica Typhimurium 14028	C57BL/6 mouse	i.m., 50 µg OMV on day 0, 5 µg OMV on days 21 and 42	Detected IgG against CPS	Sokaribo et al. (2021)
Salmonella enterica Typhimurium 1418 conjugated to *Streptococcus pyogenes* GAC	CD1 mouse<br><br>New Zealand White rabbit	i.p., on days 0 and 28, or s.c., on days 0 and 22 for mice<br><br>i.m., on days 0, 21 and 35 for rabbits	Detected IgG against *S. pyogenes* GAC in mice and rabbits	Palmieri et al. (2022)
Salmonella enterica Typhimurium NVGH2363	C57BL/6 mouse	s.c., 10 µg O-Ag in OMV with alum on days 0 and 70	Detected IgG and IgA against O-Ag (O:4,5)	Fiorino et al. (2021)
Salmonella enterica Typhimurium SGSC1418 expressing chimeric *Neisseria meningitidis* fHbp fused to *Escherichia coli* OmpA leader	CD1 mouse	s.c., with alum on days 0 and 28	Detected IgG against *S. enterica* Typhimurium O-Ag and *N. meningitidis* fHbp	Necchi et al. (2021)
Salmonella enterica Typhimurium χ3761 and χ9241 expressing chimeric *Streptococcus suis* SaoA	BALB/c mouse	i.p., 10 µg OMV on days 0 and 21	Detected IgG against *S. suis* SaoA	Li et al. (2023)

fused to *Escherichia coli* Lpp leader				
Salmonella enterica Typhimurium 2189<br><br>*S. enterica* Enteritidis 618	C57BL/6 mouse	s.c., 1 ng to 10 µg with alum on days 0 and 28	Detected IgG against LPS O-Ag	Micoli et al. (2018)
Salmonella enterica var. Typhimurium χ3761 (various LPS mutants)	BALB/c mouse	i.n., 20 µg of OMV or i.p., 5 µg OMV on days 0 and 21	Detected IgG and IgA against OMVs, as well as IgG against FliC	Liu et al. (2016)
Shigella boydii BCH612	Swiss webster mouse	Oral, 32 µg OMV on days 0, 7, 14 and 21	Detected IgG against IpaB, IpaC and IpaD	Mitra et al. (2012)
Shigella boydii BCH612<br><br>*Shigella dysenteriae* NT4907<br><br>*Shigella flexneri* B294, C519 and C347<br><br>*Shigella sonnei* IDH00968	Swiss albino mouse	Oral, 50 µg of mixed OMVs on days 0, 7, 14 and 21	Detected IgG against OMVs, putatively identified VirG, IpaB, IpaC, IpaD and OmpA using western blot	Mitra et al. (2013)
Shigella flexneri<br><br>*Shigella sonnei* (Multispecies)	CD1 mouse	s.c. or i.p., 1-500 ng of O-Ag in OMVs on days 0 and 28	Detected IgG against LPS and O-Ag	Arato et al. (2021); Mancini et al. (2023); Raso et al. (2020); Richardson et al. (2021)
Shigella flexneri 2a 2457A<br><br>*Shigella sonnei* ATCC 25931	CD1 mouse	s.c., 500 ng of O-Ag in OMVs on days 0 and 28	Detected IgG against LPS and O-Ag for *Shigella* and *Salmonella*	Gasperini et al. (2021b)

Salmonella Typhimurium 1418				
Shigella sonnei 53G<br><br>*Salmonella Typhimurium* 2192	C3H, BALB/c, and C57BL/6 mice	i.m., 100 ng of O-Ag in OMVs on days 0 and 28	Detected IgGs against LPS and O-Ag for *Shigella* and *Salmonella*	Piccioli et al. (2022)
Shigella sonnei 53G	CD1 mouse	i.n., 10 µg of OMV on days 0 and 28	Detected IgG against *S. sonnei* LPS, co-immunoprecipitated OmpA, OmpC, BamA, BamB, BamC and BamD	Mancini et al. (2021)
Shigella sonnei 53G NVGH1790 genetically modified by deletion of the late acyltransferase genes htrB and integration of *Escherichia coli* nadA and nadB genes, resulting in expression of penta-acylated LPS (O-Ag)	BALB/c mouse<br><br>New Zealand White rabbit	i.p., 29-238 µg of OMV with 2% Alhydrogel on days 0 and 21 for mice<br><br>i.m., with 100 µg, i.d., with 10 µg, or i.n., with 80 µg of OMV - Alhydrogel at weeks 0, 2, 4 and 6 for rabbits	Detected IgG against *S. sonnei* LPS in both animals	Gerke et al. (2015)
Shigella sonnei 53G NVGH1790 genetically modified by deletion of the late acyltransferase genes htrB and integration of *Escherichia coli* nadA and nadB genes, resulting in expression of penta-acylated LPS (O-Ag)	Human	1-100 µg OMV with alum on days 0, 28 and for some day 56	Detected IgG against LPS	Frenck et al. (2021); Launay et al. (2017); Launay et al. (2019); Micoli et al. (2021); Obiero et al. (2017)
Shigella sonnei 53G<br><br>*Shigella flexneri* 2a 2457T, 1b NCTC5 and 3a NCTC6885	CD1 mouse	i.p., 2.3-600 ng O-Ag in OMVs on day 0	Detected IgG against *S. sonnei* LPS and *S. flexneri* O-Ag	Necchi et al. (2023)

Staphylococcus aureus JE2	Swiss webster mouse	s.c., 5 µg of OMV on days 0, 14 and 28	Detected IgG against HLA and LukE	Wang et al. (2018)
Streptococcus mutans UA159	BALB/c mouse	i.n., 0.5 or 2.5 µg OMVs with 5 µg poly(I:C) adjuvant	Detected IgG and IgA against OMVs, partial validation of serum against GtfC	Nakamura et al. (2020)
Vibrio cholerae O1 Ogawa and O1 Inaba	BALB/c mouse	i.n., 25 µg of OMV on days 0, 14 and 18	Detected IgG1 against O1 Ogawa + Inaba OMVs, IgG1 and IgA against LPS	Bishop et al. (2010)
Vibrio cholerae AC53 expressing *Escherichia coli* H10407-S CfaB or chimeric FlaA-FliC fusion<br><br>Enterotoxigenic *E. coli* H10407-S	BALB/c mouse	i.n., 25 µg of a single OMV or 12.5 µg of each of two OMV types combined on days 0, 14 and 28	Detected IgG, IgM and IgA responses against *V. cholerae* LPS as well as recombinant *E. coli* FliC and CfaB in animals immunized with *V. cholerae* OMV, and *E. coli* LPS in animals immunized with *E. coli* H10407-S OMV	Leitner et al. (2015)
Vibrio cholerae C7258, El Tor Ogawa C7258	BALB/c mouse	i.n., OMVs containing 25 µg of LPS at days 0 and 28	Detected IgG against Ogawa LPS	Perez et al. (2009)
Vibrio cholerae Inaba O1 ATCC 39315, chitosan encapsulated	BALB/c mouse	i.d., 10 µg of OMV on day 0 or orally, 50 µg of OMV on day 0, i.p., 10 µg of OMV on days 10, 20 and 30	Detected IgG against OMVs, LPS and CTB, detected sIgA against OMV	Adriani et al. (2018)
Vibrio cholerae O1	BALB/c mouse	i.n., 25 µg of OMVs on days 0, 14 and 28	Detected IgG to OMVs, LPS and TcpA	Leitner et al. (2013)
Vibrio cholerae AC53 expressing *Escherichia coli* PhoA	BALB/c mouse	i.n., 0.0025 - 25 µg of OMV at days 0, 14 and 28	Detected IgG against *E. coli* PhoA and OMVs	Schild et al. (2009)
Yersinia pseudotuberculosis YptbS44 *expressing*	BALB/c mouse	i.m., 50 µg OMV on days 0 and 21	Detected IgG and IgM against *P. aeruginosa* PcrV-HitA fusion antigen	Li et al. (2021)

chimeric Pseudomonas aeruginosa PcrV-HitA fused to a beta-lactamase signal sequence				

Abbreviations: ALO: anthrolysin O; BCG: bacillus Calmette-Guérin; BSA: bovine serum albumin; CEACAM: carcinoembryonic antigen-related cell adhesion molecule; CPS: capsular polysaccharide; CTB: cholera toxin B; EF: edema factor; ELISA: enzyme-linked immunosorbent assay; F1: F1 capsular antigen; FCA: Freund's complete adjuvant; FHA: filamentous hemagglutinin; fHbp: factor H binding protein; FIA: Freund's incomplete adjuvant; GAC: group A carbohydrate; GFP: green fluorescent protein; HBHA: heparin-binding hemagglutinin adhesin; HLA: alpha-hemolysin; Hup: haem-utilization protein; i.d.: intradermal; IgA: immunoglobulin A; IgG: immunoglobulin G; IgM: immunoglobulin M; IgY: immunoglobulin Y; i.n.: intranasal; i.m.: intramuscular; i.p.: intraperitoneal; Kdo: 3-deoxy-d-manno-octulosonic acid; LAM: lipoarabinomannan; LF: lethal factor; Lf: flocculating units; LKT: leukotoxin; LOS: lipooligosaccharide; LNnT: lacto-N-neotetraose; LPS: lipopolysaccharide; LT: heat-labile toxin; MPL+TDM: monophosphoryl lipid A + trehalose dicorynomycolate adjuvant; MOMP: major outer membrane protein; NHBA: Neisserial heparin binding antigen; O-Ag: O-antigen; OMP: outer membrane protein; OMV: outer membrane vesicle; PA: protective antigen; pAb: polyclonal antibody; PLY: pneumolysin; PNAG: poly-N-acetyl-D-glucosamine; PRN: pertactin; PSA: polysaccharide A; PT: pertussis toxin; Sbi: *Staphylococcus aureus* binder of IgG; s.c.: subcutaneous; sIgA: secretory immunoglobulin A; SLO: streptolysin O; SpyCEP: *Streptococcus pyogenes* cell-envelope proteinase; SSA-1: serotype 1-specific antigen; wtOMV: wild-type outer membrane vesicle

References

Adriani R, Mousavi Gargari SL, Nazarian S, Sarvary S, Noroozi N (2018) Immunogenicity of *Vibrio cholerae* outer membrane vesicles secreted at various environmental conditions. Vaccine 36:322-330

Alfini R, Brunelli B, Bartolini E, Carducci M, Luzzi E, Ferlicca F, Buccato S, Galli B, Lo Surdo P, Scarselli M, Romagnoli G, Cartocci E, Maione D, Savino S, Necchi F, Delany I, Micoli F (2022) Investigating the role of antigen orientation on the immune response elicited by *Neisseria meningitidis* factor H binding protein on GMMA. Vaccines (Basel) 10:1182

Arato V, Oldrini D, Massai L, Gasperini G, Necchi F, Micoli F (2021) Impact of O-acetylation on *S. flexneri* 1b and 2a O-antigen immunogenicity in mice. Microorganisms 9:2360

Arigita C, Kersten GF, Hazendonk T, Hennink WE, Crommelin DJ, Jiskoot W (2003) Restored functional immunogenicity of purified meningococcal PorA by incorporation into liposomes. Vaccine 21:950-960

Ayalew S, Confer AW, Shrestha B, Wilson AE, Montelongo M (2013) Proteomic analysis and immunogenicity of *Mannheimia haemolytica* vesicles. Clin Vaccine Immunol 20:191-196

Bai D, Nakao R, Ito A, Uematsu H, Senpuku H (2015) Immunoreactive antigens recognized in serum samples from mice intranasally immunized with *Porphyromonas gingivalis* outer membrane vesicles. Pathog Dis 73:ftu006

Bartolini E, Ianni E, Frigimelica E, Petracca R, Galli G, Berlanda Scorza F, Norais N, Laera D, Giusti F, Pierleoni A, Donati M, Cevenini R, Finco O, Grandi G, Grifantini R (2013) Recombinant outer membrane vesicles carrying *Chlamydia muridarum* HtrA induce antibodies that neutralize chlamydial infection *in vitro*. J Extracell Vesicles 2:20181

Bash MC, Lynn F, Concepcion NF, Tappero JW, Carlone GM, Frasch CE (2000) Genetic and immunologic characterization of a novel serotype 4, 15 strain of N*eisseria meningitidis*. FEMS Immunol Med Microbiol 29:169-176

Beernink PT, Ispasanie E, Lewis LA, Ram S, Moe GR, Granoff DM (2019a) A meningococcal native outer membrane vesicle vaccine with attenuated endotoxin and overexpressed factor H binding protein elicits gonococcal bactericidal antibodies. J Infect Dis 219:1130-1137

Beernink PT, Shaughnessy J, Pajon R, Braga EM, Ram S, Granoff DM (2012) The effect of human factor H on immunogenicity of meningococcal native outer membrane vesicle vaccines with over-expressed factor H binding protein. PLoS Pathog 8:e1002688

Beernink PT, Vianzon V, Lewis LA, Moe GR, Granoff DM (2019b) A meningococcal outer membrane vesicle vaccine with overexpressed mutant fHbp elicits higher protective antibody responses in infant rhesus macaques than a licensed serogroup B vaccine. mBio 10:e01231-19

Bishop AL, Schild S, Patimalla B, Klein B, Camilli A (2010) Mucosal immunization with *Vibrio cholerae* outer membrane vesicles provides maternal protection mediated by antilipopolysaccharide antibodies that inhibit bacterial motility. Infect Immun 78:4402-4420

Bottero D, Zurita ME, Gaillard ME, Bartel E, Vercellini C, Hozbor D (2018) Membrane vesicles derived from *Bordetella bronchiseptica*: active constituent of a new vaccine against infections caused by this pathogen. Appl Environ Microbiol 84:e01877-17

Carvalho AL, Fonseca S, Miquel-Clopes A, Cross K, Kok KS, Wegmann U, Gil-Cordoso K, Bentley EG, Al Katy SHM, Coombes JL, Kipar A, Stentz R, Stewart JP, Carding SR (2019a) Bioengineering commensal bacteria-derived outer membrane vesicles for delivery of biologics to the gastrointestinal and respiratory tract. J Extracell Vesicles 8:1632100

Carvalho AL, Miquel-Clopes A, Wegmann U, Jones E, Stentz R, Telatin A, Walker NJ, Butcher WA, Brown PJ, Holmes S, Dennis MJ, Williamson ED, Funnell SGP, Stock M, Carding SR (2019b) Use of bioengineered human commensal gut bacteria-derived microvesicles for mucosal plague vaccine delivery and immunization. Clin Exp Immunol 196:287-304

Chen L, Valentine JL, Huang CJ, Endicott CE, Moeller TD, Rasmussen JA, Fletcher JR, Boll JM, Rosenthal JA, Dobruchowska J, Wang Z, Heiss C, Azadi P, Putnam D, Trent MS, Jones BD, DeLisa MP (2016) Outer membrane vesicles displaying engineered glycotopes elicit protective antibodies. Proc Natl Acad Sci U S A 113:E3609-3618

Dalseg R, Wedege E, Holst J, Haugen IL, Hoiby EA, Haneberg B (1999) Outer membrane vesicles from group B meningococci are strongly immunogenic when given intranasally to mice. Vaccine 17:2336-2345

Daniels-Treffandier H, de Nie K, Marsay L, Dold C, Sadarangani M, Reyes-Sandoval A, Langford PR, Wyllie D, Hill F, Pollard AJ, Rollier CS (2016) Impact of reducing complement inhibitor binding on the immunogenicity of native *Neisseria meningitidis* outer membrane vesicles. PLoS One 11:e0148840

De Benedetto G, Alfini R, Cescutti P, Caboni M, Lanzilao L, Necchi F, Saul A, MacLennan CA, Rondini S, Micoli F (2017) Characterization of O-antigen delivered by Generalized Modules for Membrane Antigens (GMMA) vaccine candidates against nontyphoidal *Salmonella*. Vaccine 35:419-426

Devi SJ, Zollinger WD, Snoy PJ, Tai JY, Costantini P, Norelli F, Rappuoli R, Frasch CE (1997) Preclinical evaluation of group B *Neisseria meningitidis* and *Escherichia coli* K92 capsular polysaccharide-protein conjugate vaccines in juvenile rhesus monkeys. Infect Immun 65:1045-1052

Fantappie L, de Santis M, Chiarot E, Carboni F, Bensi G, Jousson O, Margarit I, Grandi G (2014) Antibody-mediated immunity induced by engineered *Escherichia coli* OMVs carrying heterologous antigens in their lumen. J Extracell Vesicles 3:24015

Fiorino F, Pettini E, Koeberling O, Ciabattini A, Pozzi G, Martin LB, Medaglini D (2021) Long-term anti-bacterial immunity against systemic infection by *Salmonella enterica* serovar Typhimurium elicited by a GMMA-based vaccine. Vaccines (Basel) 9:495

Frenck RW, Jr., Conti V, Ferruzzi P, Ndiaye AGW, Parker S, McNeal MM, Dickey M, Granada JP, Cilio GL, De Ryck I, Necchi F, Suvarnapunya AE, Rossi O, Acquaviva A, Chandrasekaran L, Clarkson KA, Auerbach J, Marchetti E, Kaminski RW, Micoli F, Rappuoli R, Saul A, Martin LB, Podda A (2021) Efficacy,

safety, and immunogenicity of the *Shigella sonnei* 1790GAHB GMMA candidate vaccine: results from a phase 2b randomized, placebo-controlled challenge study in adults. EClinicalMedicine 39:101076

Fukasawa LO, Gorla MC, Schenkman RP, Garcia LR, Carneiro SM, Raw I, Tanizaki MM (1999) *Neisseria meningitidis* serogroup C polysaccharide and serogroup B outer membrane vesicle conjugate as a bivalent meningococcus vaccine candidate. Vaccine 17:2951-2958

Gasperini G, Alfini R, Arato V, Mancini F, Aruta MG, Kanvatirth P, Pickard D, Necchi F, Saul A, Rossi O, Micoli F, Mastroeni P (2021a) *Salmonella* Paratyphi A outer membrane vesicles displaying Vi polysaccharide as a multivalent vaccine against enteric fever. Infect Immun 89:e00699-20

Gasperini G, Biagini M, Arato V, Gianfaldoni C, Vadi A, Norais N, Bensi G, Delany I, Pizza M, Arico B, Leuzzi R (2018) Outer membrane vesicles (OMV)-based and proteomics-driven antigen selection identifies novel factors contributing to *Bordetella pertussis* adhesion to epithelial cells. Mol Cell Proteomics 17:205-215

Gasperini G, Raso MM, Arato V, Aruta MG, Cescutti P, Necchi F, Micoli F (2021b) Effect of O-antigen chain length regulation on the immunogenicity of *Shigella* and *Salmonella* Generalized Modules for Membrane Antigens (GMMA). Int J Mol Sci 22:1309

Gerke C, Colucci AM, Giannelli C, Sanzone S, Vitali CG, Sollai L, Rossi O, Martin LB, Auerbach J, Di Cioccio V, Saul A (2015) Production of a *Shigella sonnei* vaccine based on Generalized Modules for Membrane Antigens (GMMA), 1790GAHB. PLoS One 10:e0134478

Granoff DM, Costa I, Konar M, Giuntini S, Van Rompay KK, Beernink PT (2015) Binding of complement factor H (FH) decreases protective anti-FH binding protein antibody responses of infant rhesus macaques immunized with a meningococcal serogroup B vaccine. J Infect Dis 212:784-792

Haneberg B, Dalseg R, Wedege E, Hoiby EA, Haugen IL, Oftung F, Andersen SR, Naess LM, Aase A, Michaelsen TE, Holst J (1998) Intranasal administration of a meningococcal outer membrane vesicle vaccine induces persistent local mucosal antibodies and serum antibodies with strong bactericidal activity in humans. Infect Immun 66:1334-1341

Hou VC, Koeberling O, Welsch JA, Granoff DM (2005) Protective antibody responses elicited by a meningococcal outer membrane vesicle vaccine with overexpressed genome-derived Neisserial antigen 1870. J Infect Dis 192:580-590

Howlader DR, Koley H, Sinha R, Maiti S, Bhaumik U, Mukherjee P, Dutta S (2018) Development of a novel *S.* Typhi and Paratyphi A outer membrane vesicles based bivalent vaccine against enteric fever. PLoS One 13:e0203631

Hu R, Li J, Zhao Y, Lin H, Liang L, Wang M, Liu H, Min Y, Gao Y, Yang M (2020) Exploiting bacterial outer membrane vesicles as a cross-protective vaccine candidate against avian pathogenic *Escherichia coli* (APEC). Microb Cell Fact 19:119

Huang W, Wang S, Yao Y, Xia Y, Yang X, Li K, Sun P, Liu C, Sun W, Bai H, Chu X, Li Y, Ma Y (2016) Employing *Escherichia coli*-derived outer membrane vesicles as an antigen delivery platform elicits protective immunity against *Acinetobacter baumannii* infection. Sci Rep 6:37242

Huang W, Zhang Q, Li W, Chen Y, Shu C, Li Q, Zhou J, Ye C, Bai H, Sun W, Yang X, Ma Y (2019) Anti-outer membrane vesicle antibodies increase antibiotic sensitivity of pan-drug-resistant *Acinetobacter baumannii*. Front Microbiol 10:1379

Kanojia G, Raeven RHM, van der Maas L, Bindels THE, van Riet E, Metz B, Soema PC, Ten Have R, Frijlink HW, Amorij JP, Kersten GFA (2018) Development of a thermostable spray dried outer membrane vesicle pertussis vaccine for pulmonary immunization. J Control Release 286:167-178

Keenan J, Oliaro J, Domigan N, Potter H, Aitken G, Allardyce R, Roake J (2000) Immune response to an 18-kilodalton outer membrane antigen identifies lipoprotein 20 as a *Helicobacter pylori* vaccine candidate. Infect Immun 68:3337-3343

Keiser PB, Biggs-Cicatelli S, Moran EE, Schmiel DH, Pinto VB, Burden RE, Miller LB, Moon JE, Bowden RA, Cummings JF, Zollinger WD (2011) A phase 1 study of a meningococcal native outer membrane vesicle vaccine made from a group B strain with deleted *lpxL1* and *synX*, over-expressed factor H binding protein, two PorAs and stabilized OpcA expression. Vaccine 29:1413-1420

Klouwens MJ, Salverda MLM, Trentelman JJ, Ersoz JI, Wagemakers A, Gerritzen MJH, van der Ley PA, Hovius JW (2021) Vaccination with meningococcal outer membrane vesicles carrying *Borrelia* OspA protects against experimental Lyme borreliosis. Vaccine 39:2561-2567

Koeberling O, Delany I, Granoff DM (2011a) A critical threshold of meningococcal factor H binding protein expression is required for increased breadth of protective antibodies elicited by native outer membrane vesicle vaccines. Clin Vaccine Immunol 18:736-742

Koeberling O, Giuntini S, Seubert A, Granoff DM (2009) Meningococcal outer membrane vesicle vaccines derived from mutant strains engineered to express factor H binding proteins from antigenic variant groups 1 and 2. Clin Vaccine Immunol 16:156-162

Koeberling O, Ispasanie E, Hauser J, Rossi O, Pluschke G, Caugant DA, Saul A, MacLennan CA (2014) A broadly-protective vaccine against meningococcal disease in sub-Saharan Africa based on Generalized Modules for Membrane Antigens (GMMA). Vaccine 32:2688-2695

Koeberling O, Seubert A, Granoff DM (2008) Bactericidal antibody responses elicited by a meningococcal outer membrane vesicle vaccine with overexpressed factor H-binding protein and genetically attenuated endotoxin. J Infect Dis 198:262-270

Koeberling O, Seubert A, Santos G, Colaprico A, Ugozzoli M, Donnelly J, Granoff DM (2011b) Immunogenicity of a meningococcal native outer membrane vesicle vaccine with attenuated endotoxin and over-expressed factor H binding protein in infant rhesus monkeys. Vaccine 29:4728-4734

Koeberling O, Welsch JA, Granoff DM (2007) Improved immunogenicity of a H44/76 group B outer membrane vesicle vaccine with over-expressed genome-derived Neisserial antigen 1870. Vaccine 25:1912-1920

Konig E, Gagliardi A, Riedmiller I, Andretta C, Tomasi M, Irene C, Frattini L, Zanella I, Berti F, Grandi A, Caproni E, Fantappie L, Grandi G (2021) Multi-antigen outer

membrane vesicle engineering to develop polyvalent vaccines: the *Staphylococcus aureus* case. Front Immunol 12:752168
Kuipers K, Daleke-Schermerhorn MH, Jong WS, ten Hagen-Jongman CM, van Opzeeland F, Simonetti E, Luirink J, de Jonge MI (2015) *Salmonella* outer membrane vesicles displaying high densities of pneumococcal antigen at the surface offer protection against colonization. Vaccine 33:2022-2029
Launay O, Lewis DJM, Anemona A, Loulergue P, Leahy J, Scire AS, Maugard A, Marchetti E, Zancan S, Huo Z, Rondini S, Marhaba R, Finco O, Martin LB, Auerbach J, Cohen D, Saul A, Gerke C, Podda A (2017) Safety profile and immunologic responses of a novel vaccine against *Shigella sonnei* administered intramuscularly, intradermally and intranasally: results from two parallel randomized phase 1 clinical studies in healthy adult volunteers in Europe. EBioMedicine 22:164-172
Launay O, Ndiaye AGW, Conti V, Loulergue P, Scire AS, Landre AM, Ferruzzi P, Nedjaai N, Schutte LD, Auerbach J, Marchetti E, Saul A, Martin LB, Podda A (2019) Booster vaccination with GVGH *Shigella sonnei* 1790GAHB GMMA vaccine compared to single vaccination in unvaccinated healthy European adults: results from a phase 1 clinical trial. Front Immunol 10:335
Leduc I, Connolly KL, Begum A, Underwood K, Darnell S, Shafer WM, Balthazar JT, Macintyre AN, Sempowski GD, Duncan JA, Little MB, Rahman N, Garges EC, Jerse AE (2020) The serogroup B meningococcal outer membrane vesicle-based vaccine 4CMenB induces cross-species protection against *Neisseria gonorrhoeae*. PLoS Pathog 16:e1008602
Leitner DR, Feichter S, Schild-Prufert K, Rechberger GN, Reidl J, Schild S (2013) Lipopolysaccharide modifications of a cholera vaccine candidate based on outer membrane vesicles reduce endotoxicity and reveal the major protective antigen. Infect Immun 81:2379-2393
Leitner DR, Lichtenegger S, Temel P, Zingl FG, Ratzberger D, Roier S, Schild-Prufert K, Feichter S, Reidl J, Schild S (2015) A combined vaccine approach against *Vibrio cholerae* and ETEC based on outer membrane vesicles. Front Microbiol 6:823
Li P, Wang X, Sun X, Guan Z, Sun W (2021) Outer membrane vesicles displaying a heterologous PcrV-HitA fusion antigen promote protection against pulmonary *Pseudomonas aeruginosa* infection. mSphere 6:e0069921
Li Q, Zhou G, Fei X, Tian Y, Wang S, Shi H (2023) Engineered bacterial outer membrane vesicles with lipidated heterologous antigen as an adjuvant-free vaccine platform for *Streptococcus suis*. Appl Environ Microbiol 89:e0204722
Liu Q, Liu Q, Yi J, Liang K, Liu T, Roland KL, Jiang Y, Kong Q (2016) Outer membrane vesicles derived from *Salmonella* Typhimurium mutants with truncated LPS induce cross-protective immune responses against infection of *Salmonella enterica* serovars in the mouse model. Int J Med Microbiol 306:697-706
Liu Q, Yi J, Liang K, Zhang X, Liu Q (2017) Outer membrane vesicles derived from *Salmonella enteritidis* protect against the virulent wild-type strain infection in a mouse model. J Microbiol Biotechnol 27:1519-1528
Mancini F, Alfini R, Caradonna V, Monaci V, Carducci M, Gasperini G, Piccioli D, Biagini M, Giannelli C, Rossi O, Pizza M, Micoli F (2023) Exploring the role of GMMA

components in the immunogenicity of a 4-valent vaccine against *Shigella*. Int J Mol Sci 24:2742
Mancini F, Gasperini G, Rossi O, Aruta MG, Raso MM, Alfini R, Biagini M, Necchi F, Micoli F (2021) Dissecting the contribution of O-antigen and proteins to the immunogenicity of *Shigella sonnei* generalized modules for membrane antigens (GMMA). Sci Rep 11:906
Marini A, Rossi O, Aruta MG, Micoli F, Rondini S, Guadagnuolo S, Delany I, Henderson IR, Cunningham AF, Saul A, MacLennan CA, Koeberling O (2017) Contribution of factor H-binding protein sequence to the cross-reactivity of meningococcal native outer membrane vesicle vaccines with over-expressed fHbp variant group 1. PLoS One 12:e0181508
Martin DR, Ruijne N, McCallum L, O'Hallahan J, Oster P (2006) The VR2 epitope on the PorA P1.7-2,4 protein is the major target for the immune response elicited by the strain-specific group B meningococcal vaccine MeNZB. Clin Vaccine Immunol 13:486-491
Matthias KA, Connolly KL, Begum AA, Jerse AE, Macintyre AN, Sempowski GD, Bash MC (2022) Meningococcal detoxified outer membrane vesicle vaccines enhance gonococcal clearance in a murine infection model. J Infect Dis 225:650-660
Matthias KA, Reveille A, Connolly KL, Jerse AE, Gao YS, Bash MC (2020) Deletion of major porins from meningococcal outer membrane vesicle vaccines enhances reactivity against heterologous serogroup B *Neisseria meningitidis* strains. Vaccine 38:2396-2405
McConnell MJ, Rumbo C, Bou G, Pachon J (2011) Outer membrane vesicles as an acellular vaccine against *Acinetobacter baumannii*. Vaccine 29:5705-5710
Micoli F, Alfini R, Di Benedetto R, Necchi F, Schiavo F, Mancini F, Carducci M, Palmieri E, Balocchi C, Gasperini G, Brunelli B, Costantino P, Adamo R, Piccioli D, Saul A (2020) GMMA is a versatile platform to design effective multivalent combination vaccines. Vaccines (Basel) 8:540
Micoli F, Rondini S, Alfini R, Lanzilao L, Necchi F, Negrea A, Rossi O, Brandt C, Clare S, Mastroeni P, Rappuoli R, Saul A, MacLennan CA (2018) Comparative immunogenicity and efficacy of equivalent outer membrane vesicle and glycoconjugate vaccines against nontyphoidal *Salmonella*. Proc Natl Acad Sci U S A 115:10428-10433
Micoli F, Rossi O, Conti V, Launay O, Scire AS, Aruta MG, Nakakana UN, Marchetti E, Rappuoli R, Saul A, Martin LB, Necchi F, Podda A (2021) Antibodies elicited by the *Shigella sonnei* GMMA vaccine in adults trigger complement-mediated serum bactericidal activity: results from a phase 1 dose escalation trial followed by a booster extension. Front Immunol 12:671325
Mitra S, Barman S, Nag D, Sinha R, Saha DR, Koley H (2012) Outer membrane vesicles of *Shigella boydii* type 4 induce passive immunity in neonatal mice. FEMS Immunol Med Microbiol 66:240-250
Mitra S, Chakrabarti MK, Koley H (2013) Multi-serotype outer membrane vesicles of *Shigellae* confer passive protection to the neonatal mice against shigellosis. Vaccine 31:3163-3173
Muralinath M, Kuehn MJ, Roland KL, Curtiss R, 3rd (2011) Immunization with *Salmonella enterica* serovar Typhimurium-derived outer membrane vesicles

delivering the pneumococcal protein PspA confers protection against challenge with *Streptococcus pneumoniae*. Infect Immun 79:887-894

Nagaputra JC, Rollier CS, Sadarangani M, Hoe JC, Mehta OH, Norheim G, Saleem M, Chan H, Derrick JP, Feavers I, Pollard AJ, Moxon ER (2014) *Neisseria meningitidis* native outer membrane vesicles containing different lipopolysaccharide glycoforms as adjuvants for meningococcal and nonmeningococcal antigens. Clin Vaccine Immunol 21:234-242

Nakamura T, Iwabuchi Y, Hirayama S, Narisawa N, Takenaga F, Nakao R, Senpuku H (2020) Roles of membrane vesicles from *Streptococcus mutans* for the induction of antibodies to glucosyltransferase in mucosal immunity. Microb Pathog 149:104260

Nakao R, Kobayashi H, Iwabuchi Y, Kawahara K, Hirayama S, Ramstedt M, Sasaki Y, Kataoka M, Akeda Y, Ohnishi M (2022) A highly immunogenic vaccine platform against encapsulated pathogens using chimeric probiotic *Escherichia coli* membrane vesicles. NPJ Vaccines 7:153

Necchi F, Giannelli C, Acquaviva A, Alfini R, Monaci V, Arato V, Rossi O, Micoli F (2023) From an *in vivo* to an *in vitro* relative potency (IVRP) assay to fully characterize a multicomponent O-antigen based vaccine against *Shigella*. Carbohydr Polym 314:120920

Necchi F, Stefanetti G, Alfini R, Palmieri E, Carducci M, Di Benedetto R, Schiavo F, Aruta MG, Giusti F, Ferlenghi I, Goh YS, Rondini S, Micoli F (2021) *Neisseria meningitidis* factor H binding protein surface exposure on *Salmonella* Typhimurium GMMA is critical to induce an effective immune response against both diseases. Pathogens 10:726

Nieves W, Petersen H, Judy BM, Blumentritt CA, Russell-Lodrigue K, Roy CJ, Torres AG, Morici LA (2014) A *Burkholderia pseudomallei* outer membrane vesicle vaccine provides protection against lethal sepsis. Clin Vaccine Immunol 21:747-754

Norheim G, Aase A, Caugant DA, Hoiby EA, Fritzsonn E, Tangen T, Kristiansen P, Heggelund U, Rosenqvist E (2005) Development and characterisation of outer membrane vesicle vaccines against serogroup A *Neisseria meningitidis*. Vaccine 23:3762-3774

Norheim G, Arne Hoiby E, Caugant DA, Namork E, Tangen T, Fritzsonn E, Rosenqvist E (2004) Immunogenicity and bactericidal activity in mice of an outer membrane protein vesicle vaccine against *Neisseria meningitidis* serogroup A disease. Vaccine 22:2171-2180

Norheim G, Tunheim G, Naess LM, Kristiansen PA, Caugant DA, Rosenqvist E (2012) An outer membrane vesicle vaccine for prevention of serogroup A and W-135 meningococcal disease in the African meningitis belt. Scand J Immunol 76:99-107

Noroozi N, Gargari SLM, Nazarian S, Sarvary S, Adriani RR (2018) Immunogenicity of enterotoxigenic *Escherichia coli* outer membrane vesicles encapsulated in chitosan nanoparticles. Iran J Basic Med Sci 21:284-291

O'Dwyer C A, Reddin K, Martin D, Taylor SC, Gorringe AR, Hudson MJ, Brodeur BR, Langford PR, Kroll JS (2004) Expression of heterologous antigens in commensal

Neisseria spp.: preservation of conformational epitopes with vaccine potential. Infect Immun 72:6511-6518
Obiero CW, Ndiaye AGW, Scire AS, Kaunyangi BM, Marchetti E, Gone AM, Schutte LD, Riccucci D, Auerbach J, Saul A, Martin LB, Bejon P, Njuguna P, Podda A (2017) A phase 2a randomized study to evaluate the safety and immunogenicity of the 1790GAHB generalized modules for membrane antigen vaccine against *Shigella sonnei* administered intramuscularly to adults from a shigellosis-endemic country. Front Immunol 8:1884
Pajon R, Fergus AM, Granoff DM (2013) Mutant native outer membrane vesicles combined with a serogroup A polysaccharide conjugate vaccine for prevention of meningococcal epidemics in Africa. PLoS One 8:e66536
Palmieri E, Kis Z, Ozanne J, Di Benedetto R, Ricchetti B, Massai L, Carducci M, Oldrini D, Gasperini G, Aruta MG, Rossi O, Kontoravdi C, Shah N, Mawas F, Micoli F (2022) GMMA as an alternative carrier for a glycoconjugate vaccine against group A *Streptococcus*. Vaccines (Basel) 10:1034
Peak IR, Srikhanta YN, Weynants VE, Feron C, Poolman JT, Jennings MP (2013) Evaluation of truncated NhhA protein as a candidate meningococcal vaccine antigen. PLoS One 8:e72003
Peeters CC, Claassen IJ, Schuller M, Kersten GF, van der Voort EM, Poolman JT (1999) Immunogenicity of various presentation forms of PorA outer membrane protein of *Neisseria meningitidis* in mice. Vaccine 17:2702-2712
Perez JL, Acevedo R, Callico A, Fernandez Y, Cedre B, Ano G, Gonzalez L, Falero G, Talavera A, Perez O, Garcia L (2009) A proteoliposome based formulation administered by the nasal route produces vibriocidal antibodies against El Tor Ogawa *Vibrio cholerae* O1 in BALB/c mice. Vaccine 27:205-212
Perrett KP, McVernon J, Richmond PC, Marshall H, Nissen M, August A, Percell S, Toneatto D, Nolan T (2015) Immune responses to a recombinant, four-component, meningococcal serogroup B vaccine (4CMenB) in adolescents: a phase III, randomized, multicentre, lot-to-lot consistency study. Vaccine 33:5217-5224
Petersen H, Nieves W, Russell-Lodrigue K, Roy CJ, Morici LA (2014) Evaluation of a *Burkholderia pseudomallei* outer membrane vesicle vaccine in nonhuman primates. Procedia Vaccinol 8:38-42
Pettersson A, Kortekaas J, Weynants VE, Voet P, Poolman JT, Bos MP, Tommassen J (2006) Vaccine potential of the *Neisseria meningitidis* lactoferrin-binding proteins LbpA and LbpB. Vaccine 24:3545-3557
Piccioli D, Alfini R, Monaci V, Arato V, Carducci M, Aruta MG, Rossi O, Necchi F, Anemona A, Bartolini E, Micoli F (2022) Antigen presentation by follicular dendritic cells to cognate B cells is pivotal for Generalised Modules for Membrane Antigens (GMMA) immunogenicity. Vaccine 40:6305-6314
Piccioli D, Buricchi F, Bacconi M, Bechi N, Galli B, Ferlicca F, Luzzi E, Cartocci E, Marchi S, Romagnoli G, Alfini R, Di Benedetto R, Gallorini S, Savino S, Brunelli B, Bartolini E, Micoli F (2023) Enhanced systemic humoral immune response induced in mice by Generalized Modules for Membrane Antigens (GMMA) is associated with affinity maturation and isotype switching. Vaccines (Basel) 11:1219

Prados-Rosales R, Carreno LJ, Batista-Gonzalez A, Baena A, Venkataswamy MM, Xu J, Yu X, Wallstrom G, Magee DM, LaBaer J, Achkar JM, Jacobs WR, Jr., Chan J, Porcelli SA, Casadevall A (2014) Mycobacterial membrane vesicles administered systemically in mice induce a protective immune response to surface compartments of *Mycobacterium tuberculosis*. mBio 5:e01921-14

Price NL, Goyette-Desjardins G, Nothaft H, Valguarnera E, Szymanski CM, Segura M, Feldman MF (2016) Glycoengineered outer membrane vesicles: a novel platform for bacterial vaccines. Sci Rep 6:24931

Quakyi EK, Frasch CE, Buller N, Tsai CM (1999) Immunization with meningococcal outer-membrane protein vesicles containing lipooligosaccharide protects mice against lethal experimental group B *Neisseria meningitidis* infection and septic shock. J Infect Dis 180:747-754

Raeven RH, Brummelman J, Pennings JL, van der Maas L, Tilstra W, Helm K, van Riet E, Jiskoot W, van Els CA, Han WG, Kersten GF, Metz B (2016) *Bordetella pertussis* outer membrane vesicle vaccine confers equal efficacy in mice with milder inflammatory responses compared to a whole-cell vaccine. Sci Rep 6:38240

Raeven RH, Brummelman J, Pennings JLA, van der Maas L, Helm K, Tilstra W, van der Ark A, Sloots A, van der Ley P, van Eden W, Jiskoot W, van Riet E, van Els CA, Kersten GF, Han WG, Metz B (2018) Molecular and cellular signatures underlying superior immunity against *Bordetella pertussis* upon pulmonary vaccination. Mucosal Immunol 11:979-993

Raeven RH, van der Maas L, Tilstra W, Uittenbogaard JP, Bindels TH, Kuipers B, van der Ark A, Pennings JL, van Riet E, Jiskoot W, Kersten GF, Metz B (2015) Immunoproteomic profiling of *Bordetella pertussis* outer membrane vesicle vaccine reveals broad and balanced humoral immunogenicity. J Proteome Res 14:2929-2942

Raeven RHM, Rockx-Brouwer D, Kanojia G, van der Maas L, Bindels THE, Ten Have R, van Riet E, Metz B, Kersten GFA (2020) Intranasal immunization with outer membrane vesicle pertussis vaccine confers broad protection through mucosal IgA and Th17 responses. Sci Rep 10:7396

Raso MM, Gasperini G, Alfini R, Schiavo F, Aruta MG, Carducci M, Forgione MC, Martini S, Cescutti P, Necchi F, Micoli F (2020) GMMA and glycoconjugate approaches compared in mice for the development of a vaccine against *Shigella flexneri* serotype 6. Vaccines (Basel) 8:160

Reyes F, Tirado Y, Puig A, Borrero R, Reyes G, Fernandez S, Perez JL, Kadir R, Zayas C, Norazmi MN, Sarmiento ME, Acosta A (2013) Immunogenicity and cross-reactivity against *Mycobacterium tuberculosis* of proteoliposomes derived from *Mycobacterium bovis* BCG. BMC Immunol 14 Suppl 1:S7

Richardson NI, Ravenscroft N, Arato V, Oldrini D, Micoli F, Kuttel MM (2021) Conformational and immunogenicity studies of the *Shigella flexneri* serogroup 6 O-antigen: the effect of O-acetylation. Vaccines (Basel) 9:432

Rivera J, Cordero RJ, Nakouzi AS, Frases S, Nicola A, Casadevall A (2010) *Bacillus anthracis* produces membrane-derived vesicles containing biologically active toxins. Proc Natl Acad Sci U S A 107:19002-19007

Roier S, Fenninger JC, Leitner DR, Rechberger GN, Reidl J, Schild S (2013) Immunogenicity of *Pasteurella multocida* and *Mannheimia haemolytica* outer membrane vesicles. Int J Med Microbiol 303:247-256

Roier S, Leitner DR, Iwashkiw J, Schild-Prufert K, Feldman MF, Krohne G, Reidl J, Schild S (2012) Intranasal immunization with nontypeable *Haemophilus influenzae* outer membrane vesicles induces cross-protective immunity in mice. PLoS One 7:e42664

Rojas-Lopez M, Martinelli M, Brandi V, Jubelin G, Polticelli F, Soriani M, Pizza M, Desvaux M, Rosini R (2019) Identification of lipid A deacylase as a novel, highly conserved and protective antigen against enterohemorrhagic *Escherichia coli*. Sci Rep 9:17014

Romeu B, Lastre M, Garcia L, Cedre B, Mandariote A, Farinas M, Oliva R, Rosenqvist E, Perez O (2014) Combined meningococcal serogroup A and W135 outer-membrane vesicles activate cell-mediated immunity and long-term memory responses against non-covalent capsular polysaccharide A. Immunol Res 58:75-85

Roy K, Hamilton DJ, Munson GP, Fleckenstein JM (2011) Outer membrane vesicles induce immune responses to virulence proteins and protect against colonization by enterotoxigenic *Escherichia coli*. Clin Vaccine Immunol 18:1803-1808

Salverda ML, Meinderts SM, Hamstra HJ, Wagemakers A, Hovius JW, van der Ark A, Stork M, van der Ley P (2016) Surface display of a borrelial lipoprotein on meningococcal outer membrane vesicles. Vaccine 34:1025-1033

Sanders H, Norheim G, Chan H, Dold C, Vipond C, Derrick JP, Pollard AJ, Maiden MCJ, Feavers IM (2015) FetA antibodies induced by an outer membrane vesicle vaccine derived from a serogroup B meningococcal isolate with constitutive FetA expression. PLoS One 10:e0140345

Schager AE, Dominguez-Medina CC, Necchi F, Micoli F, Goh YS, Goodall M, Flores-Langarica A, Bobat S, Cook CNL, Arcuri M, Marini A, King LDW, Morris FC, Anderson G, Toellner KM, Henderson IR, Lopez-Macias C, MacLennan CA, Cunningham AF (2018) IgG responses to porins and lipopolysaccharide within an outer membrane-based vaccine against nontyphoidal *Salmonella* develop at discordant rates. mBio 9:e02379-17

Schild S, Nelson EJ, Bishop AL, Camilli A (2009) Characterization of *Vibrio cholerae* outer membrane vesicles as a candidate vaccine for cholera. Infect Immun 77:472-484

Shoemaker DR, Saunders NB, Brandt BL, Moran EE, Laclair AD, Zollinger WD (2005) Intranasal delivery of group B meningococcal native outer membrane vesicle vaccine induces local mucosal and serum bactericidal antibody responses in rabbits. Infect Immun 73:5031-5038

Sokaribo AS, Perera SR, Sereggela Z, Krochak R, Balezantis LR, Xing X, Lam S, Deck W, Attah-Poku S, Abbott DW, Tamuly S, White AP (2021) A GMMA-CPS-based vaccine for non-Typhoidal *Salmonella*. Vaccines (Basel) 9:165

Stevenson TC, Cywes-Bentley C, Moeller TD, Weyant KB, Putnam D, Chang YF, Jones BD, Pier GB, DeLisa MP (2018) Immunization with outer membrane vesicles displaying conserved surface polysaccharide antigen elicits broadly antimicrobial antibodies. Proc Natl Acad Sci U S A 115:E3106-3115

Sun J, Lin X, He Y, Zhang B, Zhou N, Huang JD (2023) A bacterial outer membrane vesicle-based click vaccine elicits potent immune response against *Staphylococcus aureus* in mice. Front Immunol 14:1088501

Troncoso G, Sanchez S, Kolberg J, Rosenqvist E, Veiga M, Ferreiros CM, Criado M (2001) Analysis of the expression of the putatively virulence-associated neisserial protein RmpM (class 4) in commensal *Neisseria* and *Moraxella catarrhalis* strains. FEMS Microbiol Lett 199:171-176

Trzewikoswki de Lima G, Rodrigues TS, Portilho AI, Correa VA, Gaspar EB, De Gaspari E (2020) Immune responses of meningococcal B outer membrane vesicles in middle-aged mice. Pathog Dis 78:ftaa028

Viviani V, Fantoni A, Tomei S, Marchi S, Luzzi E, Bodini M, Muzzi A, Giuliani MM, Maione D, Derrick JP, Delany I, Pizza M, Biolchi A, Bartolini E (2023) OpcA and PorB are novel bactericidal antigens of the 4CMenB vaccine in mice and humans. NPJ Vaccines 8:54

Wang H, Liang K, Kong Q, Liu Q (2019) Immunization with outer membrane vesicles of avian pathogenic *Escherichia coli* O78 induces protective immunity in chickens. Vet Microbiol 236:108367

Wang X, Thompson CD, Weidenmaier C, Lee JC (2018) Release of *Staphylococcus aureus* extracellular vesicles and their application as a vaccine platform. Nat Commun 9:1379

Wedege E, Bolstad K, Aase A, Herstad TK, McCallum L, Rosenqvist E, Oster P, Martin D (2007) Functional and specific antibody responses in adult volunteers in New Zealand who were given one of two different meningococcal serogroup B outer membrane vesicle vaccines. Clin Vaccine Immunol 14:830-838

Wedege E, Froholm LO (1986) Human antibody response to a group B serotype 2a meningococcal vaccine determined by immunoblotting. Infect Immun 51:571-578

Wedege E, Kuipers B, Bolstad K, van Dijken H, Froholm LO, Vermont C, Caugant DA, van den Dobbelsteen G (2003) Antibody specificities and effect of meningococcal carriage in Icelandic teenagers receiving the Norwegian serogroup B outer membrane vesicle vaccine. Infect Immun 71:3775-3781

Weyant KB, Oloyede A, Pal S, Liao J, Jesus MR, Jaroentomeechai T, Moeller TD, Hoang-Phou S, Gilmore SF, Singh R, Pan DC, Putnam D, Locher C, de la Maza LM, Coleman MA, DeLisa MP (2023) A modular vaccine platform enabled by decoration of bacterial outer membrane vesicles with biotinylated antigens. Nat Commun 14:464

Weynants V, Denoel P, Devos N, Janssens D, Feron C, Goraj K, Momin P, Monnom D, Tans C, Vandercammen A, Wauters F, Poolman JT (2009) Genetically modified L3,7 and L2 lipooligosaccharides from *Neisseria meningitidis* serogroup B confer a broad cross-bactericidal response. Infect Immun 77:2084-2093

Williams JN, Weynants V, Poolman JT, Heckels JE, Christodoulides M (2014) Immuno-proteomic analysis of human immune responses to experimental *Neisseria meningitidis* outer membrane vesicle vaccines identifies potential cross-reactive antigens. Vaccine 32:1280-1286

Zhang L, Wen Z, Lin J, Xu H, Herbert P, Wang XM, Mehl JT, Ahl PL, Dieter L, Russell R, Kosinski MJ, Przysiecki CT (2016) Improving the immunogenicity of a trivalent

Neisseria meningitidis native outer membrane vesicle vaccine by genetic modification. Vaccine 34:4250-4256
Zhang X, Yang F, Zou J, Wu W, Jing H, Gou Q, Li H, Gu J, Zou Q, Zhang J (2018) Immunization with *Pseudomonas aeruginosa* outer membrane vesicles stimulates protective immunity in mice. Vaccine 36:1047-1054
